# Land Cover Change in Colombia: Surprising Forest Recovery Trends between 2001 and 2010

**DOI:** 10.1371/journal.pone.0043943

**Published:** 2012-08-29

**Authors:** Ana María Sánchez-Cuervo, T. Mitchell Aide, Matthew L. Clark, Andrés Etter

**Affiliations:** 1 Department of Biology, University of Puerto Rico, San Juan, Puerto Rico, United States of America; 2 Department of Geography and Global Studies, Sonoma State University, Rohnert Park, California, United States of America; 3 Departamento de Ecología y Territorio, Universidad Javeriana, Bogotá D.C., Colombia; DOE Pacific Northwest National Laboratory, United States of America

## Abstract

**Background:**

Monitoring land change at multiple spatial scales is essential for identifying hotspots of change, and for developing and implementing policies for conserving biodiversity and habitats. In the high diversity country of Colombia, these types of analyses are difficult because there is no consistent wall-to-wall, multi-temporal dataset for land-use and land-cover change.

**Methodology/Principal Findings:**

To address this problem, we mapped annual land-use and land-cover from 2001 to 2010 in Colombia using MODIS (250 m) products coupled with reference data from high spatial resolution imagery (QuickBird) in Google Earth. We used QuickBird imagery to visually interpret percent cover of eight land cover classes used for classifier training and accuracy assessment. Based on these maps we evaluated land cover change at four spatial scales country, biome, ecoregion, and municipality. Of the 1,117 municipalities, 820 had a net gain in woody vegetation (28,092 km^2^) while 264 had a net loss (11,129 km^2^), which resulted in a net gain of 16,963 km^2^ in woody vegetation at the national scale. Woody regrowth mainly occurred in areas previously classified as mixed woody/plantation rather than agriculture/herbaceous. The majority of this gain occurred in the Moist Forest biome, within the montane forest ecoregions, while the greatest loss of woody vegetation occurred in the Llanos and Apure-Villavicencio ecoregions.

**Conclusions:**

The unexpected forest recovery trend, particularly in the Andes, provides an opportunity to expand current protected areas and to promote habitat connectivity. Furthermore, ecoregions with intense land conversion (e.g. Northern Andean Páramo) and ecoregions under-represented in the protected area network (e.g. Llanos, Apure-Villavicencio Dry forest, and Magdalena-Urabá Moist forest ecoregions) should be considered for new protected areas.

## Introduction

Land cover change is the main cause of deterioration in ecological systems at local to global scales [1], [2]. Land-use/land-cover (LULC) research has mainly focused on forest conversion (deforestation) because of its impacts on global and regional climate change [2]–[5], soil degradation [6], loss of biodiversity [7], [8], and goods and services provided by natural systems [9], [10]. Consequently, knowledge of drivers, patterns, and rates of deforestation has been increasing rapidly, yet many information gaps still exist. For example, the extent of deforestation in many tropical countries is not based on current assessments, most lagging 5–10 years. Furthermore, LULC research has not fully considered other land transitions such as forest regrowth (reforestation) despite gathering evidence that there is a worldwide reforestation trend [1], [11]–[14]. According to The Food and Agriculture Organization of the United Nations (FAO), many areas of secondary forest are projected to increase, especially in the tropics [15]. In contrast, in many other areas deforestation is expected to increase (e.g. arc of deforestation in Brazil) due to regional and global factors, such as population growth and demand for food and commodities [16]. However, little is known about the spatial distribution and interactions of the process of reforestation and deforestation across tropical countries. It is important that LULC research focuses on joint analysis of gains and losses of forest area, because both processes can occur at broad spatial scales that encompass a range of environmental and socioeconomic conditions; and ultimately, the dynamics and type of forests undergoing change have serious implications for carbon sequestration, reduction in carbon dioxide emissions, biodiversity, and soil conservation.

Monitoring forest cover at the national level is essential for developing and implementing appropriate biodiversity conservation and carbon emission reduction policies [17]. The successful design and execution of these policies depends on the accuracy of forest cover estimates through consistent methodologies that follow a comparable classification scheme [17], [18]. Typically, forest cover assessments have been made using sensors with spatial resolution between 300 and 1,000 m and mostly at larger extents [19], [20]. There have also been efforts to map forest cover using sensors (e.g. Landsat, CBERS, and SPOT HVR) with finer spatial resolution (less than 100 m) [17], [21], [22] but these analyses are typically for sub-national regions. Nevertheless, land cover mapping at the national scale using higher resolution data has major limitations because of difficulties in getting cloud-free images, low temporal resolution, or high cost, and image gaps in the case of Landsat 7 [23]. Some developing countries have produced regional forest monitoring programs (e.g. India and Brazil), including data from their own national satellites with high resolution images from 20 to 70 m (see [21], [24]. However, implementing systematic forest assessments such as those in India and Brazil in other developing countries is difficult because of the limitations in technical infrastructure, expertise, and data collection costs [17]. The Moderate Resolution Imaging Spectroradiometer (MODIS) satellite data products are reliable and useful tools for monitoring land change in developing countries. Although MODIS has a minimum spatial resolution of 250 m, advantages include: high temporal resolution (i.e., daily) of imagery which can be composited to reduce cloud coverage and rapid data availability at no cost. These characteristics allow a complete LULC mapping not only at the global and national scales, but regional and sub-regional scales as well [18], [19], [25].

Colombia is one of the most biodiverse countries on Earth [26] especially in the categories of plants, mammals, reptiles, amphibians, and birds [27], [28]. Colombia also has one of the largest continuous forest areas in the tropics, covering at least 49% of the national land territory [25]. Despite its high biodiversity and natural resources, there is no consistent multi-temporal dataset of LULC change for Colombia. Forest cover assessments are mainly done by two official organizations, the IDEAM (Instituto de Hidrología, Meteorología y Estudios Ambientales) and the IAvH (Instituto de Investigación de Recursos Biológicos Alexander von Humboldt). These organizations provide maps and reports at regional to national scales based on remote sensing products from MODIS and Landsat sensors, but ground-based forest inventories have not been made at the national scale. Another source of information is FAO, which estimates forest cover every five to ten years. The most recent FAO estimates in 2010 are based on 2002 maps provided by IDEAM [29], but FAO adjusts IDEAM data with their own methodology to standardized forest assessment in multiple countries. At regional and local scales, government agencies (e.g. Corporaciones Autónomas Regionales), non-government organizations (e.g. Fundación Natura), and local and foreign universities also have collected LULC data. Unfortunately, each organization uses their own mapping approaches and different spatio-temporal scales, which makes it difficult to compare LULC data among studies, regions, and years.

**Figure 1:**
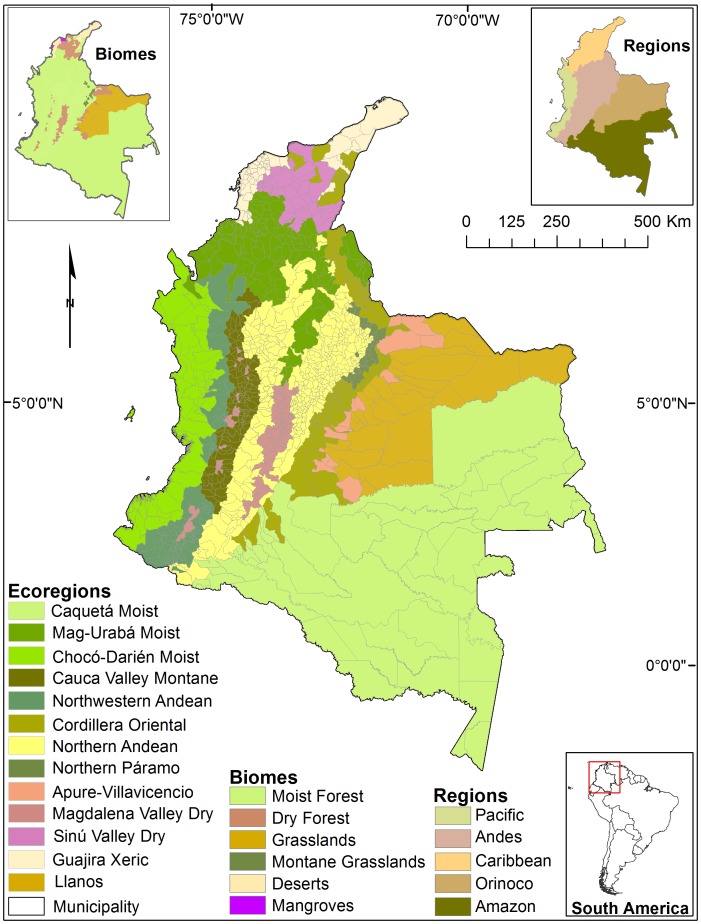
Map of the 13 ecoregions and 1117 municipalities in Colombia. Insert shows the distribution of the six biomes, and the five regions.

Land transformation is not homogeneous in Colombia, but rather varies greatly among its different ecological and political regions [26] (Figure 1). The Andes and the Caribbean regions have been the most impacted as part of the early colonization process (after 1500 AD; [30]) that severely affected its biodiversity and natural resources. However, since 1900 forest clearing has concentrated in the eastern lowlands, mainly in the Amazon and Orinoco regions [31]. Forest cover transformation in Colombia usually begins with the clearing of small areas used for subsistence agriculture, later these areas are often replaced by pastures for livestock grazing; and many of these areas are transformed in to mechanized agriculture (e.g. rice; [32]). Over the years, many such areas have been abandoned due to loss of soil productivity [32], [33], rural-urban migration, technology improvement, and globalization of markets [34]; these processes promote forest recovery, but in some cases these abandoned lands continue in a degraded state [35]. Nevertheless, there is a lack of recent information about how LULC varies across the country and its regions, and between different ecosystems. Thus, there is a need for evaluating land change at multiple spatio-temporal scales using a consistent methodology across the various ecological and socioeconomic gradients of Colombia.

The purpose of the present study is to assess land change from 2001 to 2010 in Colombia, with a focus on three objectives: (1) determine how land change varies at the country, biome, ecoregion, and municipality scales; (2) identify and analyze the spatial distribution of areas experiencing significant land change; and, (3) discuss the implications of our findings for land use and conservation planning in Colombia. This research was based on a novel method for mapping LULC annually, which coupled MODIS (250 m) products with reference data interpreted from high spatial resolution imagery (QuickBird) in Google Earth that allows us to quantify land change at multiple spatial scales.

## Methods

### Study Area

Colombia is located in the northwestern part of South America, bordered by the Caribbean Sea to the north and the Pacific Ocean to the west, and occupying an area of 1.1 million km^2^. Colombia has about 45.4 million people and an average population density of 40.1 people per km2 (see http://www.dane.gov.co). Differences in elevation and latitude produce large climatic variation across the country. For example, there are dramatic differences in annual precipitation, ranging from 350 mm (Guajira peninsula) to 12,000 mm (Pacific lowlands). Consequently, the combination of different climates, elevation ranges, and geographic location have allowed the development of a high diversity of habitats and species richness in Colombia, as well as an array of land uses.

Colombia can be divided into five continental regions (Andean, Caribbean, Pacific, Orinoco, and Amazon; Figure 1), 26 ecoregions, and 63 ecosystems [26]. These regions have remarkable biogeographic, socio-cultural, economic, and demographic differences. Consequently, LULC across the country has undergone distinct land transitions in the different regions. The Andean region is composed of three mountain ranges (Western, Central, and Eastern) that sustain montane ecosystems with multiple vegetation types. Pasturelands are the dominant land cover in the region (24%) compared with croplands (19%). The Caribbean region is characterized by xerophytic and subxerophytic vegetation types that correspond to arid and semiarid lowland areas. Lands in the Caribbean are mainly used for cattle ranching (48%) and another considerable fraction for agriculture (14%). The Pacific region contains a dense coastal lowland rain forest, where croplands cover a greatest area (10%) compared with pasturelands which cover less than 2%. In the Orinoco region (usually referred to as the Llanos), pasturelands (86%) and croplands (3%) have increased rapidly since the 1980’s. Finally, the Amazon, the largest and least transformed region of the country, is mostly covered by tropical rainforests; however, previous studies have estimated that deforestation has converted about 6% of forests into pasturelands, and less than 1% into legal and illegal croplands [36].

In this study, municipalities (second administrative level) were the main unit of analysis. According to the National Administrative Department of Statistics (DANE) data, the number of municipalities in Colombia was 1,100 in 2007. However, we included 1,097 municipalities instead of 1,100 because three municipalities were created after the last census in 2005. We also included 20 *areas no municipalizadas* or *corregimientos* (name of the third administrative level in Colombia) because they occupied a large area (almost 190,000 km^2^) in the southern portion of the country (Amazonas, Guainía and Vaupés departments). All analyses were thus performed on 1,117 municipalities or study units.

### Land-use/land-cover Mapping

Our LULC classification methodology generally follows those first outlined by [18] and modified for continental-scale mapping in [37]. Here we summarize the three main steps that pertain to the Colombian national maps used in our analysis:

1. Google Earth reference data collection (>10,000 samples): reference data for classifier training and accuracy assessment were collected with human interpretation of high-resolution imagery in Google Earth (GE, http://earth.google.com) mainly from Digital Globe’s QuickBird satellite (http://www.digitalglobe.com) spanning 2001 to 2010 [38]. Visual interpretation methods followed those in [18], [38] and were performed by the authors (AMSC, M.A, and M.C) and student technicians. Samples were 250×250-m areas placed manually across the tropical and subtropical moist broadleaf forests, tropical and subtropical dry broadleaf forests, and tropical and subtropical grasslands, savannas and shrublands biomes [39], which covered Colombia and extended into neighboring countries [38] (Figure S1). Samples were located with both random sampling and stratified random sampling, which included areas with mixed cover types, and samples well within patches of homogeneous cover, and no two samples were closer than 1,000 m apart [38]. Prior to interpretation, sample centers were snapped to the closest satellite image pixel (MODIS). Each sample class was assigned the year of the image and the percent cover of seven cover classes was visually interpreted: *woody* (woody vegetation including trees and shrubs); *herb* (herbaceous vegetation); *ag* (agriculture); *plant* (plantations); *built* (built-up areas); *bare* (bare areas); and *water*. If two interpreters agreed on the majority cover and GE image year of a sample, then their percent cover estimates were averaged. If the interpreters disagreed on the majority cover or year (mostly cover), then an “expert” (author) estimated the final class cover and recorded the year [38]. Samples were assigned to a class if the cover in this class was ≥80%. Samples with 20–80% *woody*, with a *bare*, *herb* or *ag* component <80% were assigned to a *mixed woody* class.
2. Satellite imagery used in classification: we used the MODIS MOD13Q1 Vegetation Indices 250 m product (Collection 5) for LULC classification [18], [37]. The product is a 16-day composite of the highest-quality pixels from daily images and includes the Enhanced Vegetation Index (EVI), red, near infrared (NIR), and mid-infrared (MIR) reflectance and pixel reliability [40]. Twenty-three samples were available per year, with data available from 2001 to present. All MODIS scenes were reprojected from their native Sinusoidal projection to the Interrupted Goode Homolosine projection (sphere radius of 6,378,137.0 m) using nearest-neighbor resampling. The original cell size of 231.7 meters was maintained in the reprojection. For each pixel, we calculated the mean, standard deviation, minimum, maximum, and range statistics for EVI, and red, NIR and MIR reflectance values for calendar years 2001 to 2010. Statistics were calculated for all 12 months, 2 six-month periods, and 3 four-month periods. The MOD13Q1 pixel reliability layer was used to remove all unreliable samples (value = 3) prior to calculating statistics. If fewer than three samples were available for a statistics temporal window for a given year, then the statistics for that window were given null values.
3. Mapping LULC with the Random Forest classifier: we mapped LULC with the Random Forests (RF) tree-based classifier [41] following methods in [37]. An advantage of the RF classifier is that it provides an assessment of error with “out-of-bag” (OOB) samples, a form of multi-fold cross-validation [18], [41]. These data can be used to calculate an error matrix, an unbiased estimate of accuracy, rather than withholding samples in an independent test dataset [18], [41]. RF classifier was implemented using R (v. 2.12.2; [42]) and the *randomForest* package (v. 4.6−2; [43]) with 1999 decision trees, a minimum of 5 samples in terminal nodes (nodesize = 5), and sqrt(*p*) as number of variables randomly sampled as candidates at each split, where *p* is number of variables (mtry = default). Predictor variables were MODIS-based 4-, 6- and 12-month statistics for EVI, red, NIR and MIR, and were extracted for the year corresponding to the QuickBird image year (range 2001 to 2010 [38]) for each GE reference sample. We trained four separate RF based on samples in separate biomes with boundaries defined by municipalities. The tropical and subtropical moist broadleaf forests biome was split to include an Amazon basin section and a coastal lowlands section, while the desert and xeric shrublands biome was combined with the tropical and subtropical dry broadleaf forest biome [37], [38] (Figure S1). An initial RF for a biome was generated with the reference data class and MODIS predictor variables from that biome. The *outlier* function in randomForest was used to eliminate samples with an outlier metric greater than 10, and a final RF was generated from the remaining samples, leaving 10,143 of 10,622 (96%) samples for training the final RF (Table 1). We used R and the RGDAL library to apply the RF objects to every pixel in MODIS tiles covering the zone-biome region for each year, 2001 to 2010. For a given year, if a pixel had valid 4-, 6- and 12-month statistics, then the class was assigned based on the initial RF; a secondary RF based on just 12-month statistics was applied to pixels that had only valid 12-month statistics; and, the pixel was assigned a No Data value if it had no valid predictor variables (e.g., areas with persistent cloud cover, beach/water interfaces along coasts). On average each annual map had 0.14%±0.09% of the area covering Colombia mapped as No Data. Pixels with ≥4 No Data values over 10 years were set to a null value and excluded from our maps, as these were unreliable areas for mapping – mostly coastal areas in Colombia. The four separate maps were then mosaicked and reclassified (post-classification) by grouping *ag* and *herb*, *mixed woody* and *plant*, and *built* and *bare*. The combining of classes into a five-class scheme helped reduce inter-class confusion and increase map accuracy while still allowing us to focus on major trajectories of change in *woody* vegetation. Based on the OOB statistics, the final five-class maps had an average overall accuracy of 87.4% (±4.3%), with non-water average producer’s accuracies ranging from 36.3% (*mixed woody/plant*) to 96.9% (*woody*) and user’s accuracies ranging from 72.5% (*mixed woody/plant*) to 89.4% (*woody*) (Table 2). The five-class LULC map was then summarized for the 1,117 municipalities.

**Table 1:** Mapping regions and total sample counts used in each separate Random Forest (n = 4).

Geographic region	Ag	Bare	Built	Herb	Mixed woody	Plant	Woody	Water	Total
	Tropical and Subtropical Moist Broadleaf Forests (TSMBF)								
Amazon/Chocó	44	75	58	792	379	86	2,794	823	5,051
	334	9	131	327	151	77	797	309	2,135
	Tropical and Subtropical Dry Broadleaf Forests (TSDBF)								
Northern S.America	279	75	147	380	187	96	422	239	1,825
	Tropical and Subtropical Grasslands, Savannas, Shrublands (TSGSS)								
Llanos	201	32	42	378	116	42	167	154	1,132

These include only samples filtered by the Random Forest outlier removal step (Biomes follow Olson et al. 2001).

**Table 2:** Classification accuracy assessment.

			Producer’s Accuracy (%)					User’s Accuracy (%)				
Biomes	Samples	Overall (%)	Ag/Herb	Bare/Built	Mixed woody/plant	Woody	Water	Ag/Herb	Bare/Built	Mixed woody/plant	Woody	Water
Moist Forest^1^	5,051	92.2	86.5	64.7	49.7	100.0	99.9	78.7	86.9	73.6	97.7	95.8
Moist Forest^2^	2,135	89.2	90.5	91.4	33.3	99.6	100.0	83.5	89.5	71.0	92.6	99.0
Dry Forest^3^	1,825	82.0	87.3	90.1	32.5	92.4	100.0	78.6	82.6	68.7	81.6	100.0
Grasslands^4^	1,132	86.1	97.4	67.6	29.7	95.8	100.0	83.9	89.3	77.0	86.0	98.1
Total/Avg	10,143	87.4	90.4	78.4	36.3	96.9	99.9	81.1	87.0	72.5	89.4	98.2

**Biome description:**

1 Tropical and Subtropical Moist Broadleaf Forest (Amazon basin section).

2 Tropical and Subtropical Moist Broadleaf Forest (Coastal lowlands section).

3 Tropical and Subtropical Dry Broadleaf Forest.

4 Tropical and Subtropical Grasslands, Savannas and Shrublands.

### Land Change Dynamics

To describe the patterns of land change, for the three most important vegetation classes (*woody, mixed woody/plant,* and *ag/herb*) within each municipality, we analyzed the trends performing a linear regression of cover area (dependent variable) against time (independent variable, each of the 10 years between 2001 and 2010). If more than 1% of the total municipality area had pixels mapped as No Data for a given year, then the land cover data for that year were removed from the regression. To determine the strength of this linear relationship we used Pearson’s correlation coefficient (R), where positive values of R represent an increase in a LULC cover and negative values of R represent a decrease. We used this approach to standardize land change through time due to outliers or missing data in any given year, and the use of R for trends allows us to compare municipalities, which can vary in size from 17,6 km^2^ to 65,568 km^2^. In addition, this trend analysis takes advantage of the ten years of data, and it is not based on just two points in time. Municipalities with significant changes in any cover had *p*≤0.05. All analyses incorporating absolute area were performed using estimates based on the each municipality’s regression model, rather than the raw area data used to fit the model.

We calculated the net change in cover (km^2^) of the three classes between 2001 and 2010 considering four scales: country, biome, ecoregion, and municipality. Biome and ecoregion scales were established following the World Wildlife Fund biome and ecoregion framework [39]. We clustered municipalities into the six major biomes and 25 ecoregions that were described for Colombia (Table S1). Municipalities present in more than one biome and ecoregion were classified as the unit with the greatest area in each municipality. We included tropical and subtropical moist broadleaf forest (Moist Forest), tropical and subtropical dry broadleaf forest (Dry Forest), tropical and subtropical grassland, savanna and shrubland (Grassland), Montane Grassland and Shrubland (Montane Grassland), Desert and Xeric Shrubland (Desert), and Mangrove (Mangrove) biomes. We reduced the 25 ecoregions to 13 because some ecoregions were represented by only one or a few municipalities (Figure 1). For example, Western Ecuador Moist Forest (NT0178) was present in only one municipality (Tumaco). Therefore, this municipality was aggregated to the largest and closest ecoregion (Chocó-Darién Moist Forest/NT0115), which also contained similar environmental characteristics. We performed a Mann-Whitney test in R (v. 2.12.2; [42]) to determine if there was a significant difference in the size of municipalities that gained or lost *woody* vegetation.

## Results

### Land-use/land-cover Change from 2001 to 2010

**Figure 2:**
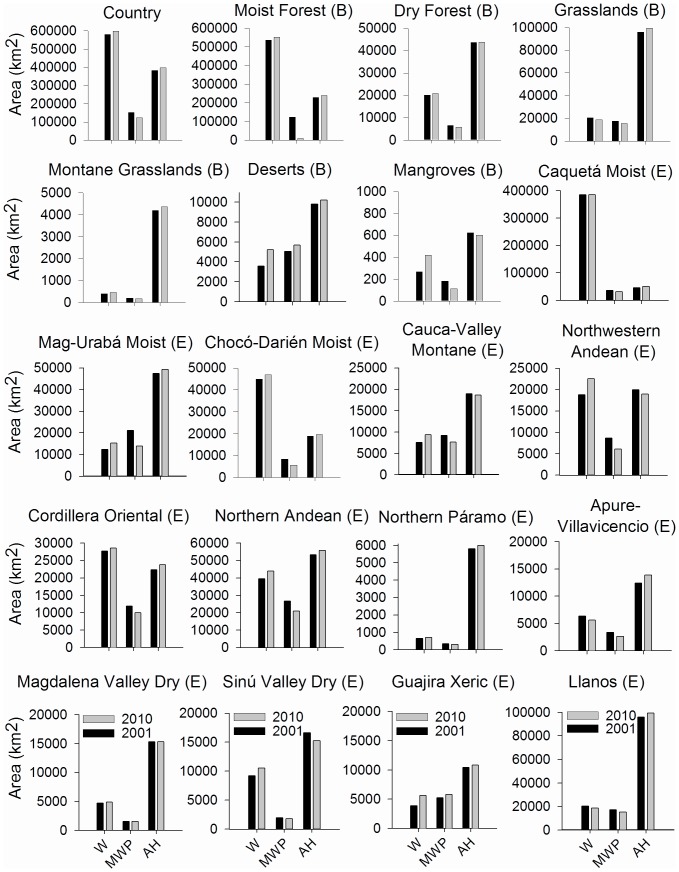
Absolute area of *woody* vegetation (W), *mixed woody/plant* (MWP), and *ag/herb* (AH) from 2001 to 2010 at the country, biome (B) and ecoregion (E) scales. These estimates are based on estimates from municipality-scale regression models and include all municipalities.

At the country level, *woody* vegetation was the most predominant land cover (Figure 2). *Woody* cover increased from 580,420 km^2^ in 2001 to 597,383 km^2^ in 2010, with a net gain of 16,963 km^2^. *Ag/herb* class also increased from 383,097 km^2^ to 397,741 km^2^ with a net gain of 14,644 km^2^. In contrast, *mixed woody/plant* decreased from 151,930 km^2^ in 2001 to 122,648 km^2^ in 2010, with a net loss of 29,282 km^2^.

At the biome level, *woody* cover only decreased in the Grasslands biome (1,636 km^2^), while it increased in the other five biomes, from 16,077 km^2^ in the Moist Forest, to 57 km^2^ in the Montane Grasslands (Figure 2). The *mixed woody/plant* class in turn, increased only in the Desert biome (621 km^2^), while there was a large decrease in the Moist Forest biome (27,181 km^2^). The *ag/herb* cover only decreased slightly in Mangroves (21 km^2^), while increasing in the rest of biomes from 10,652 km^2^ in the Moist Forest to 58 km^2^ in the Dry Forest.

In 2001 and 2010, *woody* vegetation was the dominant cover in four ecoregions, while *ag/herb* vegetation was the dominant cover in nine ecoregions (Figure 2). Land cover change varied greatly among ecoregions. *Woody* vegetation decreased only in Apure-Villavicencio and Llanos ecoregions with a reduction of 691 km^2^ and 1,636 km^2^, respectively. In the other eleven ecoregions *woody* vegetation increased, seven of which had a net gain of more than 1,200 km^2^. The net gain varied from 4,535 km^2^ in the Northern Andean forests to 63 km^2^ in the Northern Páramo ecoregions. The Caquetá Moist forest ecoregion, the largest ecoregion in Colombia (472,066 km^2^) also had a small increase in woody cover (144 km^2^) when compared with the rest of the ecoregions. *Mixed woody/plant* class increased only in the Guajira Xeric ecoregion (552 km^2^), and decreased in more than 1,500 km^2^ in eight ecoregions. *Mixed woody/plant* net loss varied between 7,147 km^2^ in the Magdalena-Urabá Moist forest and 9 km^2^ in the Magdalena Valley Dry forest ecoregions. The *ag/herb* vegetation mainly decreased in the Sinú-Valley Dry forest (1,347 km^2^), the Northwestern Andean (969 km^2^), and the Cauca-Valley Montane forest (310 km^2^). The net gain in *ag/herb* vegetation varied between 5,256 km^2^ in the Caquetá Moist forest and 25 km^2^ in the Magdalena-Valley Dry forest.

**Figure 3:**
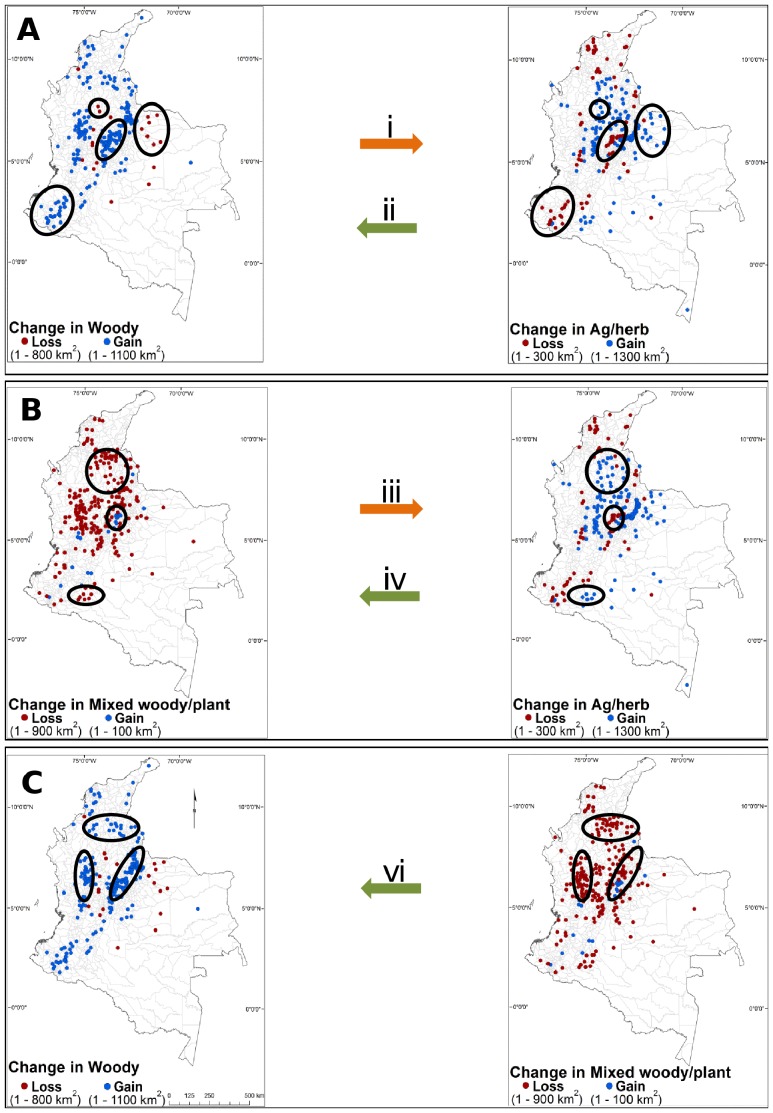
Areas of significant change in land cover. Transitions between A) *woody* vegetation and a*g/herb*; B) *mixed woody/plant* and *ag/herb*; C) *woody* vegetation and *mixed woody/plant* are shown. Red and blue dots represent municipalities with significant loss and gain in cover area (km^2^), respectively. Black ovals represent prominent clusters of land cover change. Orange and green arrows present deforestation and reforestation transitions, respectively. Land transitions (i–vi) discussed in the text.

At the municipality level, *woody* vegetation increased in 73% (820) of the municipalities with a net gain of 28,092 km^2^, and decreased in 24% (264) of the municipalities with a net loss of 11,129 km^2^ (Table S2). In contrast, the *mixed woody/plant* class increased in 31% (347) of the municipalities with a net gain of 5,199 km^2^, while it decreased in 68% (762) of municipalities with a net loss of 34,481 km^2^. For the *ag/herb* class, the number of municipalities gaining (53%; 587) and losing (47%; 526) cover was similar, but the area gained was almost double (28,345 km^2^) of that lost (13,701 km^2^). We also found that 21% (232) of the municipalities showed significant change in *woody* vegetation during the last decade. This percentage was similar for *mixed woody/plant* (23%; 254) and for *ag/herb* (20%; 225). If we only considered municipalities with significant changes during the last decade, we detected a close correspondence between loss and gain of *woody* vegetation, *mixed woody/plant,* and *ag/herb* classes (Figure 3). Examples of this dynamic include: i) areas where *woody* vegetation was transformed into *ag/herb* in the southern part of the Magdalena Medio and the Llanos piedmont regions; ii) transitions from *ag/herb* vegetation to *woody* vegetation were located in western Cundiboyacense highplain, and between Nudo de los Pastos and the Macizo Colombiano (Figure 3A); iii) transitions from *mixed woody/plant* to *ag/herb* vegetation appeared in the Magdalena Medio and the Alto Caquetá regions; iv) transitions from *ag/herb* vegetation to *mixed woody/plant* were located only in the north of the Cundiboyacense highplain (Figure 3B); v) transitions from *woody* to *mixed woody/plant* was not common; and, vi) transitions from *mixed woody/plant* to *woody* vegetation were concentrated in the Catatumbo and to the north of the Magdalena Medio, as well as to the north of the central and western Andean mountain ranges (Figure 3C). The average size of the municipalities that significantly gained or lost *woody* vegetation was 688 km^2^ and 3,113 km^2^, respectively, and this difference was significant (Mann-Whitney U = 3.6, *p* = 0.0003).

Finally, to determine the hotspots of *woody* vegetation change we selected the top 10 municipalities with the greatest net gain or loss in *woody* cover (Table 3). The top 10 municipalities with the greatest *woody* vegetation gain account for 14% of the total *woody* increase, and 42% of the increase when only considering municipalities with a significant change in *woody* vegetation. Interestingly, Cumaribo, the largest municipality in Colombia, accounted for almost 4% of total increase in *woody* vegetation and 11% considering municipalities with a significant gain. The 10 municipalities with the greatest *woody* vegetation loss account for 27% of total decrease, and 91% of municipalities with a significant loss in *woody* cover. Municipalities showing the greatest net gain were located primarily in the Magdalena-Urabá Moist forest and Chocó-Darién Moist forest, while those with the greatest net loss were located mainly in the Llanos, Apure-Villavicencio, and Northern Andes.

**Table 3:** Top ten municipalities with the greatest net gain (+) and net loss (−) of *woody* vegetation between 2001 and 2010.

Municipality	State	Ecoregion	Municipality Area(km^2^)	Net Change (km^2^)	Woody (R)	(p-value)	% Change
Cumaribo	Vichada	Caquetá Moist	65,568	+1,065	0.79	0.005	3
Tibú	N. Santander	Mag-Urabá Moist	2,680	+638	0.87	0.0008	45
Uribia	La Guajira	Guajira Xeric	7,890	+589	0.67	0.03	2,148
Maguí	Nariño	Chocó-Darién Moist	1,634	+394	0.66	0.03	62
Medio Baudó	Chocó	Chocó-Darién Moist	1,428	+344	0.63	0.04	56
Lloró	Chocó	Chocó-Darién Moist	802	+322	0.74	0.05	108
Sardinata	N. Santander	Mag-Urabá Moist	1,454	+211	0.78	0.007	26
Rioviejo	Bolívar	Mag-Urabá Moist	1,284	+178	0.64	0.04	40
San Benito Abad	Sucre	Mag-Urabá Moist	1,520	+164	0.83	0.002	447
Tiquisio	Bolívar	Mag-Urabá Moist	773	+134	0.62	0.05	52
La Macarena	Meta	Caquetá Moist	10,756	−712	−0.62	0.05	−17
Arauquita	Arauca	Apure-Villavicencio	3,218	−471	−0.63	0.04	−29
Tame	Arauca	Apure-Villavicencio	5,433	−409	−0.74	0.01	−17
Remedios	Antioquia	Northern Andean	2,045	−384	−0.87	0.001	−27
Mapiripán	Meta	Llanos	12,018	−359	−0.75	0.01	−5
Puerto Gaitán	Meta	Llanos	17,397	−199	−0.83	0.002	−10
Fortul	Arauca	Cordillera Oriental	1,067	−126	−0.84	0.03	−24
Trinidad	Casanare	Llanos	2,973	−121	−0.74	0.01	−26
San Luis de Palenque	Casanare	Llanos	3,005	−104	−0.61	0.05	−27
Segovia	Antioquia	Northern Andean	1,154	−103	−0.83	0.002	−10

Columns show area, net change, correlation coefficient (R), *p-*value, and the percentage of change of the top ten municipalities with greatest net gain and loss between 2001 and 2010.

## Discussion

### Patterns of Land Cover Change at the Country Level

Our results show that during the last decade, land change in Colombia has been characterized by an unexpected net gain in *woody* cover, increasing by 16,963 km^2^ or 3% of its initial area in 2001. In contrast, previous literature has highlighted dramatic forest loss at the national [22] and regional scales [32], [44]. *Woody* cover as well as *ag/herb* classes expanded mostly at the expense of the *mixed woody/plant* class at the national and municipality levels. At first glance, it appears that *woody* regrowth results from secondary forest/shrub recovery rather than recently abandoned agricultural areas. Forest regrowth at the national scale is consistent with the general reforestation trends in Europe, the U.S.A. [45], and in other Latin American countries such as Ecuador, the Dominican Republic, Puerto Rico, Costa Rica [11]–[14]. However, secondary vegetation regrowth in Colombia might be the effect of land abandonment resulting from armed conflicts and economic development experienced during the last 10–20 years [46]. Land abandonment of rural areas began in the early 1990s when the Colombian government implemented an economic liberalization model, and it continued in the late 1990s as a result of the intensification of internal conflicts. The effects of these conflicts and the associated political decisions have been documented for the Caquetá region [47]. Although the amount of *woody* vegetation gained was almost three times higher than the amount of forest lost, it is clear that deforestation continues. Extensive *woody* cover losses occurred in municipalities principally to the southwest of Magdalena Medio (e.g. Segovia and Remedios) and in the Llanos regions (e.g. San Luis de Palenque, Tame), where 3,000 km^2^ of *woody* vegetation were converted to croplands and pastures. Deforestation in these areas is related to gold mining and oil exploitation activities, and agricultural expansion. For example, in the Magdalena Medio region *woody* vegetation has been cleared for small-scale agriculture and timber extraction by miners since the 1990s [48]. In the Llanos, the construction of the Villanueva-Yopal road and the road infrastructure to aid oil exploration has stimulated the expansion of trading, cattle, and agriculture. For example, rice cultivation has increased from 1,300 km^2^ in 2001 to 1,800 km^2^ in 2009 [49]. The decrease in *woody* vegetation in these regions affects areas of global importance for biodiversity such as Serranía de San Lucas located to the south of Magdalena Medio [48] and along the Andean foothills in the Llanos.

Overall, we report a forest area of 580,420 km^2^ in 2001 which is lower than the 617,328 km^2^ and 615,090 km^2^ (for the year 2000) reported by IDEAM and FAO, respectively (Table 4). Additionally, we reported an increase in 2005 and 2010, which contrasts with the large forest decrease reported by IDEAM and the slight decrease reported by FAO. In contrast, our estimates are in agreement with MOD44B Vegetation Continuous Field (VCF-using an 80% forest crown cover; [50]) results which also reported a forest area increase. However, we estimated a net gain (16,963 km^2^ from 2001 to 2010) which is much more conservative than the gains (83,515 km^2^ from 2000 to 2005) estimated by MOD44B (VCF). These differences can be attributed to the use of different MODIS image inputs, spatial resolution (ours 250 m vs. VCF 500 m) and processing methods, as well as differences in forest (*woody* vegetation) definition. For example, VCF includes plantations in forest cover. FAO defines “forest” as lands of more than 0.5 ha covered by tress over 5-m height, 10% crown cover, and include areas with native and plantation trees [29]. IDEAM classifies “forest” as lands more than 1 ha, trees over 5-m height, with 30% crown cover, that also includes shrubs, palms, and bamboo, but not tree plantation [22]. In contrast, our definition of forest or “*woody*” vegetation differs from the former definitions because we include trees and shrubs, (i.e., no height requirement) with ≥80% cover. Consequently, our definition of *mixed woody* vegetation (20–80% woody) combined with plantations is more comparable to FAO’s and IDEAM’s definition of “forest”. In addition, FAO and IDEAM estimates showed the same deforestation trend because their definitions of forest are somewhat similar and FAO results are typically based on existing maps provided by IDEAM [29]. Nevertheless, IDEAM maps in 2000, 2005, and 2010 lacked information for approximately 8% of Colombia due to cloud coverage. Thus, conclusions drawn from these maps could be misleading in either direction with regard to forest cover. These areas without information from IDEAM were scattered across the country, particularly in the north portion of the Pacific region and areas spread throughout the Andes and the Amazon regions where cloud cover is high, and where at the same time we found the largest net gain in *woody* vegetation.

**Table 4:** A comparison of four estimates of *woody* vegetation class at the national scale.

			Woody vegetation area (km^2^)			
Year	This study (W)‡	This study (W+MWP)†	IDEAM^1^	FAO^2^	MOD44B^3^ (VCF 500 m)^‡^	MOD44B^3^ (VCF 500 m)±
2000	n.a.	n.a	617,328	615,090	269,195	820,392
2001	580,420	732,350	n.a.	n.a.	298,170	839,487
2005	587,953	726,817	602,063	610,040	352,710	832,261
2008	593,611	722,723	n.a.	n.a.	n.a.	n.a.
2010	597,383	720,031	586,336	604,990	n.a.	n.a.

‡ Data including only *woody* vegetation.

† Data including *woody* vegetatio*n*+*mixed woody/plant.*

‡ Forest cover (80 crown cover).

± Forest cover (25 crown cover).

**Sources:**

1 Cabrera E, Vargas DM, Galindo G, García MC, Ordoñez MF, et al. (2011).

2 FAO (2010) Global Forest Resources Assessment 2010.

3 Hansen M, DeFries R, Townshend JR, Carroll M, Dimiceli C, et al. (2006).

In general, our methodology to map annual LULC in Colombia, which combined MODIS products, Google Earth reference data, and Random Forest classifier [37], provides a consistent classification scheme at multiple spatial-temporal scales. The high accuracy values we obtained demonstrate the robustness of the mapping method and the reliability of our LULC maps which have several advantages with respect to previous maps, including: (1) quantification of both deforestation and reforestation patterns across the country at multiple spatial scales; (2) using Google Earth reference data collection for classifier training and accuracy assessment (rather than ground-based reference data collection) which provides us a fast and inexpensive way to acquire reference data across the whole country, a large part of which is difficult to access; (3) use of temporally-composited MODIS data, which greatly reduces the amount of pixels adversely affected by cloud coverage and thus allows wall-to-wall LULC change monitoring; and, (4) leveraging 10 years of annual LULC area at municipality level to better estimate 2001 to 2010 net change, thus reducing the influence of climate fluctuations or other factors that could bias analyses based on just two years of data, and to determine which municipalities had significant increases or decreases in area while normalizing differences in municipality area.

We acknowledge that there are two potential caveats to our study. First, MODIS pixels will not detect small-scale changes (e.g. slash and burn agriculture, <5 ha) due its lower spatial resolution. However, the accumulative change from the small-scale conversion can be captured by our 10-year trend analysis based on the aggregation of all pixels within a municipality. Although Landsat provides higher spatial resolution which facilitates detection of small-changes, there are major gaps due to clouds cover that make it difficult to map the whole country using Landsat imagery. Second, using reference data from QuickBird imagery in Google Earth could include interpretation and spatial error. For example, visual interpretation of some land cover classes in Google Earth is difficult, and therefore, our cover classes were very general. Even though our classes were relatively easy to identify, interpreters sometimes disagreed on *ag* and *herb* samples for which an expert determined the final class label. Additionally, spatial error can be the result of terrain distortions especially in QuickBird images that have not been orthorectified. However, it has been shown that QuickBird scenes are very accurate with an average error of 10 m of disagreement between ground control points and GE QuickBird images [18].

### Potential Factors Explaining Woody Vegetation Recovery

There are three potential factors that could explain the increase in *woody* vegetation observed in our LULC maps. The first possible explanation could be an increase in oil palm plantations. Oil palm plantations have expanded rapidly since the 1990s when Colombia initiated its economic liberalization model [51]. According to the The National Federation of Oil Palm Growers (Fedepalma), palm plantations for oil extraction increased from 180 km^2^ in the 1960s to almost 3,600 km^2^ in 2010– mainly in the Meta, Casanare, Cesar, Magdalena, Bolívar, Cundinamarca, Santander, Norte de Santander, and Nariño departments. Nevertheless, we do not believe that oil palm plantations are an important component of the *woody* recovery we described. First, we classified plantations separate from *woody* vegetation. Second, municipalities identified by IGAC [52] as having large areas of oil palm plantations do not coincide with the municipalities we identified as important areas of reforestation. In fact, our results showed that from 2006 to 2008, 76% of the municipalities had net gain in *woody* vegetation (total of 4,740 km^2^). However, during the same period of time, only 7% of the municipalities had a net gain in palm oil plantations (362 km^2^; [52]). In addition, taking into account the ten municipalities with the greatest net gain in *woody* vegetation from 2006 to 2008, only Tibú (net gain of 142 km^2^) and Riohacha (net gain of 81 km^2^) have oil palm plantations (net gain of 50 and 3 km^2^, respectively; [52]).

A second factor that could explain *woody* recovery is coca crops eradication programs. At the national scale, coca cultivation area dropped from 1,448 km^2^ in 2001 to 618 km^2^ in 2010 [53]. Eradication programs, both manually and by aerial spraying, have been implemented intensively in several localized areas of lowland forests in the Moist Forest biome. Eight of the top 10 municipalities with the greatest net gain in *woody* vegetation cultivated coca in 2001 (189 km^2^; [53]). By 2010, the area of coca in these municipalities declined to 58 km^2^. The majority of this decline occurred in Cumaribo and Tibú municipalities, which lost 128 km^2^ of coca plantations between 2001 and 2010. It is possible that we are detecting the first stages of natural regeneration (i.e. shrubs) following the eradication of these illicit crops.

The third potential factor that could explain the increase in *woody* cover, particularly in seasonal forests (i.e. dry forests), is related to the inter-annual variation in precipitation. In these areas, anomalous rainfall years (e.g. La Niña events) could change the vegetation greenness trends detected by MODIS sensors. This phenomenon could change *woody* vegetation to *mixed woody* and vice versa, especially for pixels near the 80% decision threshold. However, we attempted to minimize this effect using regression models by municipality, capturing 10-years of real trends in vegetation dynamics. It would be desirable to use high resolution data such as Landsat to evaluate the accuracy in our LULC maps and to verify observed land change; however, on an annual basis it is difficult to obtain the necessary temporal data for the entire country for detecting any climatic anomalies.

In summary, the national assessment of land cover change in Colombia indicates that *woody* vegetation gains occur in small municipalities and exceed *woody* vegetation losses occurring in large municipalities. This scale of analysis gives a valuable general overview of current land change, but it can mask *woody* vegetation losses in some areas. National scale analysis does not take into account intrinsic differences (e.g. socioeconomic, demographic, and biophysical) among regions which can promote different land cover patterns and dynamics. Therefore, by examining data at the biome and ecoregion scales it is possible to decipher where and to what extent changes in *woody* vegetation are occurring, and to better understand the underlying environmental and social drivers of this change.

### Patterns of Land Cover Change at the Biome and Ecoregion Scales

#### Moist forest biome

This biome accounts for 86% of the total increase in *woody* cover. It is the largest biome in Colombia (Table S1) consisting of seven ecoregions which contain both montane forest (4 ecoregions) and tropical forest (3 ecoregions). This recovery occurred mostly from *mixed woody/plant*, generated in previous periods and less directly from *ag/herb* vegetation (Figure 2). The net gain in *woody* vegetation was located especially in the montane forest of the Andes mountain ranges (70%) and in the tropical forest in the Amazon and the Pacific regions (30%). Other studies have quantified forest regrowth in Colombia from secondary vegetation and abandoned pastures, particularly in the Amazon [54] and in the Andes regions [22]. This pattern of forest regrowth in the Moist Forest Biome has also been reported for Venezuela and Costa Rica [55] but, deforestation continues to be the major trend in this biome. These contrasting dynamics are driven by multiple factors including an increase in the global demand for meat (deforestation), as well as the abandonment of marginal agriculture lands and changing patterns of precipitation [55]–[57].

The four montane forest ecoregions (in the Moist Forest biome) located in the Andes mountain ranges contributed 65% of the total net gain in *woody* vegetation in Colombia, particularly in the Northern Andean (27%), the Northwestern Andean (22%), and Cauca-Valley (11%) Montane Forest ecoregions. *Woody* vegetation increases are likely explained by human causes such as the abandonment of traditional productive systems in the 1990s due to harsh environmental conditions (e.g. arid areas) and globalization processes which have promoted strong rural migration in many municipalities [58]. For example, 43% of municipalities located in the Chicamocha region (Northern Andean ecoregion) lost population between 1993 and 2005 (see http://www.dane.gov.co), and the area is now experiencing significant change from *mixed woody/plant* class to *woody* vegetation (mostly shrubland cover; Figure 3C). Similar abandonment patterns can be observed in the Cundiboyacense highplains (Northern Andean ecoregion) where many municipalities are transitioning primarily from *ag/herb* to *woody* vegetation. However, reforestation programs to protect watersheds [59] and to restore degraded areas [60] could also explain part of the *woody* increase in this area. These factors have also facilitated a significant reforestation (transitioned from *ag/herb*) of large expanses of montane forest to the south of the Northwestern Andean (between the Macizo Colombiano and Nudo de los Pastos) and to the north of the Cauca-Valley Montane forest ecoregions as well. This reforestation trend has been seen in both developed and developing countries, supporting the idea that the abandonment of less productive lands and the globalization of markets may lead to the regrowth of secondary forest [61], [62].

All three ecoregions in the Moist Forest biome experienced gains in *woody* and *ag/herb* vegetation, which transitioned from the *mixed woody/plant* vegetation class. For instance, the Magdalena-Urabá Moist Forest ecoregion showed a remarkable decrease in *mixed woody/plant* class, particularly in the Magdalena Medio region where 46% transitioned to *woody* vegetation and 44% transitioned to *ag/herb* vegetation, the remaining area transitioned to other classes (i.e. *built-up* and *bare soil*). *Woody* vegetation in the Caquetá Moist forest ecoregion seems to be stable, contributing only 0.8% of the total net gain in *woody* vegetation in Colombia. These results coincide with data from the Amazon forest, which showed forest regrowth transitioned primarily from previous croplands [44]. However, our findings contrast with the IDEAM results that include the Amazon region as one of the deforestation hotspots of the country [22]. But, if we combine our *woody* vegetation and *mixed woody/plant* classes, which is more comparable with the definition of forest used by IDEAM, we detect a loss of >5,000 km^2^. Virtually all of this change is from *mixed woody/plant* to *ag/herb*. These losses in the Caquetá Moist forest are the result of small-scale subsistence agriculture (driven by rural-rural migration [63] and illicit crops, particularly in the Alto Caquetá in the Caquetá department.

#### Grasslands biome

This is the second largest biome in Colombia and includes only the Llanos ecoregion. The Llanos is increasingly being considered as the new agricultural frontier of Colombia (see: http://webapp.ciat.cgiar.org/es/descargar/pdf/convenio_colombia_ciat.pdf). Large *woody* vegetation losses were located mainly in the central area of Casanare and the eastern area of Meta departments where an intense land conversion is associated with human population change and investments in infrastructure to support an important oil exploration activity and agricultural intensification. High rates of land conversion (towards mechanized agriculture and cattle grazing) corresponding with urban population growth and migration have been registered since the 1980s [64], a pattern that appears to continue today. Between 1993 and 2005, 92% of the municipalities gained people (see http://www.dane.gov.co) and 85% gained *ag/herb* vegetation, implying that agriculture (e.g. rice) and pastures are still expanding, as in many other regions [65], [66]. Nevertheless, the transformation of savannas in the Orinoco region, which is steadily increasing and currently a major land use change in Colombia [67], cannot be quantified with our method as it will be a transition from herbaceous vegetation to agriculture, which are both contain in our combined *ag/herb* class. Changes are only detected when the change is to perennial plantations.

#### Dry forest biome

This is the third largest biome and includes three ecoregions: Magdalena Valley, Sinú Valley, and Apure-Villavicencio Dry forest. This biome accounts for 4% of the total increase in *woody* vegetation and its recovery was the result of a transition mostly from *ag/herb* vegetation in the Sinú Valley Dry forest. From 1990 to 2003, the cotton industry decreased in the Sinú valley due to changes in pricing policies and competition with the subsidized international markets that significantly affected its area and production. Consequently, this crop had an annual loss rate of 13% of cultivated land between 1990 and 2003 [68]. The Apure-Villavicencio Dry forest ecoregion showed a decrease in *woody* vegetation as a result of agriculture and pastures expansion (as in the Llanos ecoregion). This expansion was located particularly in the foothills of the Arauca department where the majority of the population is located. According to the DANE censuses, 93% of the municipalities in the Apure-Villavicencio Dry Forest ecoregion gained people between 1993 and 2005 and 87% gained *ag/herb* vegetation. We believe that the expansion of intensive agriculture and cattle pasture will continue as a major driver of deforestation in this region.

#### Desert and mangroves biomes

These biomes only include the Guajira Xeric ecoregion which had a net gain in *woody* vegetation. These biomes account for 9% and 0.7% of the national increase in *woody* vegetation, respectively. The increase in *woody* vegetation in the Desert biome was concentrated in three municipalities that alone account for 73% of the total increase in this biome. The gain in *woody* in these municipalities could be related to a precipitation anomaly (e.g. 2009) or perhaps a problem in the classification given that the Desert biome was classified as part of the Dry Forest biome (Table 2). *Woody* regrowth (mostly shrubland cover) in the Deserts have been reported in Mexico and U.S as a result of the increase in annual precipitation and the decrease of fire and grazing, respectively [57], [69]. On the other hand, in the Mangrove biome, *woody* gains are likely the result of the implementation of conservation and management strategies of mangrove ecosystems across the country [70]. For example, the increase in *woody* vegetation was located in two municipalities (98% of the total increase in this biome) that contain the Vía Parque Isla de Salamanca protected area, which was declared a Ramsar Site in 1998 and Reserve for Humankind and the Biosphere by UNESCO in 2000 (see http://www.parquesnacionales.gov.co).

#### Montane grasslands biome

This biome includes the Northern Andean Páramo ecoregion which had a slight increase in *woody* vegetation, accounting for only 0.3% of the national increase in *woody* vegetation. The gain could be the result of regrowth in areas previously occupied by *Papaver somniferum* (poppy) plantations. Between 1993 and 2008, the area in poppy fields decreased from 75 km^2^ to 4 km^2^ across Colombia [71]. This biome has also experienced a significant increase in *ag/herb* vegetation. The gains in *ag/herb* cover were located mainly in municipalities in the Santander and Boyacá departments where potato farming and cattle grazing are important activities. In these departments, the cultivated area of potatoes increased from 380 km^2^ to 482 km^2^ between 2006 and 2008 [52] in response to the national and international demand for potato products. Since the potato is the agricultural product with highest consumption per capita in Colombia [72], its cultivation is expected to expand in the near future, adding more pressure on the Páramo ecosystems.

### Implications for Conservation Planning

The implementation of protected areas is an important mechanism to reduce forest conversion and subsequent loss of species [73]. In Colombia, 56 protected areas (12% of the national’s territory) are effectively reducing the probability of forest clearing [44]. Nevertheless, many protected areas are located in places with lower forest conversion risks [74]. Therefore, to increase the impact of conservation efforts, the Colombian protected area network should target areas with low levels of protection and high rates of land transformation. For instance, the Llanos and the Apure-Villavicencio ecoregions are underrepresented in the protected area network (see http://www.parquesnacionales.gov. co), and this is where we detected the highest rates of *woody* vegetation loss. These areas have had extensive areas of natural savanna vegetation transformed to crops and pastures during the past 20 years [64], [67]. We suggest that a primary conservation goal in Colombia should be the implementation of protected areas in these regions. The Llanos ecoregion is particularly important given its heterogeneous landscapes, its high diversity of vegetation types, and its large numbers of plants, amphibians, reptiles, and fish [75]. Not surprisingly, this ecoregion has been cataloged within the Global 200, which is a set of the most outstanding ecoregions for global conservation [75]. In addition, the Apure-Villavicencio dry forest should be taken into account in the protected areas network because it represents the transition zone between the Andean foothills and the llanos savannas where a relatively high number of plant, reptile, and bird species (including several endemics) coexist (See http://www.worldwildlife.org/wildworld/profiles/terrestrial_nt.html# tropgrass). We also documented a large decrease in *mixed woody/plant* in the Magdalena-Urabá Moist forest ecoregion, particularly in the Magdalena Medio region. The increase in agriculture and pastures combined with ongoing illegal logging activities [70], [76] have endangered a great number of native timber species (e.g. *Libidibia ebano, Cariniana pyriformis*). This region should be considered for the protected area network given that there is only one reserve (Serranía de los Yariguíes national park) in this region.

On the other hand, the recovery of *woody* vegetation in the Andes Mountain Ranges is an excellent opportunity to complement, expand and interconnect the protected areas to create a conservation network across the rural landscape mosaics in the region. A relevant area for conservation is the Northern Andean Montane Forest ecoregion, which is also included in the Global 200 [75]. In this ecoregion, the Cundiboyacense highplain had a substantial and significant gain of *woody* vegetation between 2001 and 2010. Other authors have stressed the importance of this region as a priority area for conservation due to its large areas of land transformation and large number of species at risk [74]. We also highlight that even though the Northern Andean Páramo ecoregion gained slight amounts of *woody* vegetation, the gains in the *ag/herb* class were almost three times higher than *woody* cover gains, and therefore, the Andean Páramos remains a threatened ecosystem.

Overall, the present study indicates that at the national scale, *woody* vegetation gains exceed losses between 2001 and 2010. The majority of *woody* gains occurred in the Moist Forest biome. Analysis at the ecoregion scale showed that montane forest ecoregions contributed substantially to *woody* vegetation regrowth in Colombia, while the Llanos and Apure-Villavicencio ecoregions experienced the largest *woody* losses. The gain of *woody* vegetation does not necessarily imply the recovery of the high biodiversity characteristic of the original forests in many of these regions. If these “new forests” are allowed to grow, they are likely to recover a large proportion of their biodiversity in the next 40–50 years [77]. Guiding efficient conservation actions requires a better understanding of land cover change and its drivers. Consequently, our maps and land cover trends are a baseline to evaluate the effects of environmental, socioeconomic, and demographic factors on land cover change in Colombia.

## Supporting Information

Figure S1 **Distribution of reference data points collected from Google Earth within each of the three biomes which covered Colombia and neighboring countries.** Biome description: 1. Tropical and Subtropical Moist Broadleaf Forest (Amazon basin section) 2. Tropical and Subtropical Moist Broadleaf Forest (Coastal lowlands section) 3. Tropical and Subtropical Dry Broadleaf Forest 4. Tropical and Subtropical Grasslands, Savannas and Shrublands
(TIF)

Table S1 **Major biomes and ecoregions in Colombia.** The names and area of the 6 major biomes and the 25 ecoregions in Colombia according to Olson et al. (2001). Note that the original 25 ecoregions were grouped into 13 ecoregions because some ecoregions only include one or a few municipalities. The name of the largest ecoregion was used as the name of the aggregation.
(DOCX)

Table S2 **Woody vegetation net gain and loss for all municipalities in Colombia.** Thirty three municipalities were not included because they did not have any *woody* vegetation.
(DOCX)

## References

[1] Lambin EF, Turner BL, Geist HJ, Agbola SB, Angelsen A, et al (2001) The causes of land-use and land-cover change: moving beyond the myths. Global Environmental Change-Human and Policy Dimensions 11: 261–269.

[2] Lambin EF, Geist HJ, Lepers E (2003) Dynamics of land-use and land-cover change in tropical regions. Annual Review of Environment and Resources 28: 205–241.

[3] Lambin EF (1997) Modelling and monitoring land-cover change processes in tropical regions. Progress in Physical Geography 21: 375–393.

[4] Geist HJ, Lambin EF (2002) Proximate causes and underlying driving forces of tropical deforestation. Bioscience 52: 143–150.

[5] Gash JHC, Nobre CA, Roberts JM, Victoria LM (1996) Amazonian deforestation and climate. Chichester, New York: John Wiley & Sons. 611 p.

[6] Trimble SW, Crosson P (2000) Land use - US soil erosion rates - Myth and reality. Science 289: 248–250. 17750403 10.1126/science.289.5477.248

[7] Thuiller W, Lavorel S, Araujo MB, Sykes MT, Prentice IC (2005) Climate change threats to plant diversity in Europe. Proceedings of the National Academy of Sciences of the United States of America 102: 8245–8250. 15919825 10.1073/pnas.0409902102 PMC1140480

[8] Sala OE, Chapin FS, Armesto JJ, Berlow E, Bloomfield J, et al (2000) Biodiversity - Global biodiversity scenarios for the year 2100. Science 287: 1770–1774. 10710299 10.1126/science.287.5459.1770

[9] Vitousek PM, Mooney HA, Lubchenco J, Melillo JM (1997) Human domination of Earth’s ecosystems. Science 277: 494–499.

[10] Kremen C, Williams NM, Aizen MA, Gemmill-Herren B, LeBuhn G, et al (2007) Pollination and other ecosystem services produced by mobile organisms: a conceptual framework for the effects of land-use change. Ecology Letters 10: 299–314. 17355569 10.1111/j.1461-0248.2007.01018.x

[11] Hecht SB, Kandel S, Gomes I, Cuellar N, Rosa H (2006) Globalization, forest resurgence, and environmental politics in El Salvador. World Development 34: 308–323.

[12] Lugo AE, López Marrero T, Ramos González OM, Vélez L (2004) Urbanización de los terrenos en la periferia de El Yunque. Washington, DC: USDA Forest Service.

[13] Grau HR, Aide TM, Zimmerman JK, Thomlinson JR, Helmer E, et al (2003) The ecological consequences of socioeconomic and land-use changes in postagriculture Puerto Rico. Bioscience 53: 1159–1168.

[14] Rudel TK, Bates D, Machinguiashi R (2002) A tropical forest transition? Agricultural change, out-migration, and secondary forests in the Ecuadorian Amazon. Annals of the Association of American Geographers 92: 87–102.

[15] FAO (2005) FAO Statistical database 2005. Available: http://faostat.fao.org. Accessed 2012 Feb 15.

[16] Barona E, Ramankutty N, Hyman G, Coomes OT (2010) The role of pasture and soybean in deforestation of the Brazilian Amazon. Environmental Research Letters 5.

[17] DeFries R, Achard F, Brown S, Herold M, Murdiyarso D, et al (2007) Earth observations for estimating greenhouse gas emissions from deforestation in developing countries. Environmental Science & Policy 10: 385–394.

[18] Clark ML, Aide TM, Grau HR, Riner G (2010) A scalable approach to mapping annual land-cover at 250 m using MODIS time-series data: A case study in the Dry Chaco ecoregion of South America. Remote Sensing of Environment 114: 2816–2832.

[19] Friedl MA, Sulla-Menashe D, Tan B, Schneider A, Ramankutty N, et al (2010) MODIS Collection 5 global land cover: Algorithm refinements and characterization of new datasets. Remote Sensing of Environment 114: 168–182.

[20] Bartholome E, Belward AS (2005) GLC2000: a new approach to global land cover mapping from Earth observation data. International Journal of Remote Sensing 26: 1959–1977.

[21] INPE (2005) Projeto PRODES: Monitoramento da Floresta Amazônica Brasileira por satélite. Available: http://www.obt.inpe.br/prodes/index.html. Accessed 2012 Jan 29.

[22] Cabrera E, Vargas DM, Galindo G, García MC, Ordoñez MF, et al. (2011) Memoria técnica de la cuantificación de la deforestación histórica nacional – escalas gruesa y fina. Bogotá, D.C, Colombia: Instituto de Hidrología, Meteorología y Estudios Ambientales (IDEAM). 103 p.

[23] Hansen MC, Roy DP, Lindquist E, Adusei B, Justice CO, et al (2008) A method for integrating MODIS and Landsat data for systematic monitoring of forest cover and change in the Congo Basin. Remote Sending of Environment. Remote Sending of Environment 112: 2495–2513.

[24] Forest Survey of India (2009) State of Forest Report 2009. Dehra Dun, India: Ministry of Environment and Forest, Government of India. 199 p.

[25] Achard F, Eva H, Mollicone D, Popatov P, Stibig H, et al. (2009) Detecting Intact Forest from Space: Hot Spots of loss, Deforestation and the UNFCCC. In: Wirth C, Gleixner G, Heimann M, editors. Old-Growth Forests: Function, Fate and Value. Germany: Springer-Verlag Berlin Heidelberg. 411–427.

[26] Chaves ME, Arango N, editors (1998) Informe Nacional sobre el estado de la Biodiversidad en Colombia 1997. Bogotá, DC, Colombia: Instituto de Investigación de Recursos Biológicos Alexander von Humboldt.

[27] Myers N, Mittermeier RA, Mittermeier CG, da Fonseca GAB, Kent J (2000) Biodiversity hotspots for conservation priorities. Nature 403: 853–858. 10706275 10.1038/35002501

[28] Orme CDL, Davies RG, M Burgess, F Eigenbrod, N Pickup, et al (2005) Global hotspots of species richness are not congruent with endemism or threat. Nature 436: 1016–1019. 16107848 10.1038/nature03850

[29] FAO (2010) Global Forest Resources Assessment 2010. Rome, Italy: Food and Agriculture Organization of the United Nations. 58 p.

[30] Etter A, van Wyngaarden W (2000) Patterns of landscape transformation in Colombia, with emphasis in the Andean region. Ambio 29: 432–439.

[31] Etter A, McAlpine C, Pullar D, Possingham H (2005) Modeling the age of tropical moist forest fragments in heavily-cleared lowland landscapes of Colombia. Forest Ecology and Management 208: 249–260.

[32] Etter A, McAlpine C, Pullar D, Possingham H (2006a) Modelling the conversion of Colombian lowland ecosystems since 1940: Drivers, patterns and rates. Journal of Environmental Management 79: 74–87. 16171932 10.1016/j.jenvman.2005.05.017

[33] Aide TM, Cavelier J (1994) Barriers to lowland tropical forest restoration in the Sierra Nevada de Santa Marta, Colombia. Restoration Ecology 2: 219–229.

[34] Etter A, McAlpine C, Possingham H (2008) Historical patterns and drivers of landscape change in Colombia since 1500: A regionalized spatial approach. Annals of the Association of American Geographers 98: 2–23.

[35] Gunter S, Gonzalez P, Alvarez G, Aguirre N, Palomeque X, et al (2009) Determinants for successful reforestation of abandoned pastures in the Andes: Soil conditions and vegetation cover. Forest Ecology and Management 258: 81–91.

[36] IDEAM IGAC, IAvH Invemar, Sinchi, etal. (2007) Mapa de ecosistemas continentales, costeros y marinos de Colombia (escala 1: 500.000). Bogotá, D.C, Colombia: Instituto de Hidrología, Meteorología y Estudios Ambientales (IDEAM), Instituto Geográfico Agustín Codazzi (IGAC), Instituto de Investigación de Recursos Biológicos Alexander von Humboldt (IAvH), Instituto de Investigaciones Ambientales del Pacífico Jhon von Neumann (IIAP), Instituto de Investigaciones Marinas y Costeras José Benito Vives De Andréis (Invemar) e Instituto Amazónico de Investigaciones Científicas Sinchi (Sinchi).

[37] Clark ML, Aide. TM. An analysis of decadal land change in Latin America and the Caribbean mapped from 250–m MODIS data; 2011a; 34th International Symposium on Remote Sensing of Environment; Sydney, Australia.

[38] Clark ML, Aide TM (2011b) Virtual interpretation of Earth Web-Interface Tool (VIEW-IT) for collecting land-use/land-cover reference data. Remote Sensing 3: 601–620.

[39] Olson DM, Dinerstein E, Wikramanayake ED, Burgess ND, Powell GVN, et al (2001) Terrestrial ecoregions of the worlds: A new map of life on Earth. Bioscience 51: 933–938.

[40] Huete A, Didan K, Miura T, Rodriguez E, Gao X, et al (2002) Overview of the radiometric and biophysical performance of the MODIS vegetation indices. Remote Sensing of Environment 83: 195–213.

[41] Breiman L (2001) Random Forest. Machine Learning 45: 5–32.

[42] R DCT (2011) R: A language and environment for statistical computing. R Foundation for Statistical Computing Vienna, Austria. ISBN 3–900051–07–0, URL http://www.R-project.org.

[43] Liaw A, Wiener M (2002) Classification and Regression by randomForest. R News 2: 18–22.

[44] Dávalos LM, Bejarano AC, Hall M, Correa HL, Corthals A, et al (2011) Forest and drugs: coca-driven deforestation in tropical biodiversity hotspots. Environmental Science & Technology 45: 1219–1227. 21222455 10.1021/es102373d

[45] Kauppi PE, Ausubel JH, Fang JY, Mather AS, Sedjo RA, et al (2006) Returning forests analyzed with the forest identity. Proceedings of the National Academy of Sciences of the United States of America 103: 17574–17579. 17101996 10.1073/pnas.0608343103 PMC1635979

[46] PNUD (2011) Colombia rural. Razones para la esperanza. Bogotá, D.C, Colombia: INDH PNUD. 443 p.

[47] Etter A, McAlpine C, Phinn S, Pullar D, Possingham H (2006b) Characterizing a tropical deforestation wave: the Caquetá colonization front in the Colombian Amazon. Global Change Biology 12: 1409–1420.

[48] Salaman PGW, Donegan TM (2001) Presenting the first biological assessment of Serranía de San Lucas, 1999–2001. Colombian EBA Project Report Series. Bogotá, D.C, Colombia: Fundación Proaves. 36 p.

[49] Gutierrez AN, Barón J, Roa J, Castro G, Mendoza G, et al. (2011) Dinámica del sector arrocero de los Llanos Orientales de Colombia, 1999–2011. Bogotá, D.C, Colombia: Federación Nacional de Arroceros. 159 p.

[50] Hansen M, DeFries R, Townshend JR, Carroll M, Dimiceli C, et al. (2006) Vegetation Continuous Fields MOD44B, 2001 Percent Tree Cover, Collection 4. College Park, Maryland: University of Maryland, 2001.

[51] Aguilera M (2002) Palma africana en la Costa Caribe: un semillero de empresas solidarias. Cartagena, Colombia: Centro de estudios económicos regionales, Banco de la República. 53 p.

[52] IGAC (2011) Sistema de información geográfica para la planeación y el ordenamiento territorial nacional SIGOT. Available: http://sigotn.igac.gov.co/sigotn/default.aspx. Accessed 2012 Jan 25.

[53] UNODC (2011) Monitoreo de cultivos de coca 2010. Bogotá, D.C, Colombia: United Nations Office for Drug and Crime. 116 p.

[54] Etter A, McAlpine C, Phinn S, Pullar D, Possingham H (2006c) Unplanned land clearing of Colombian rainforests: spreading like disease?. Landscape and Urban Planning 77: 240–254.

[55] Aide TM, Clark ML, Grau R, López-Carr D, Levy M, et al. (2012) Deforestation and reforestation of Latin America and the Caribbean (2001–2010). Biotropica: In press.

[56] Redo D, Aide TM, Clark ML (2012) The Relative Importance of Socio-Economic and Environmental Variables in Explaining Land Change in Bolivia, 2001–2009. Annals of the Association of American Geographers: In press.

[57] Bonilla-Moheno M, Aide TM, Clark M (2011) The influence of socioeconomic, environmental, and demographic factors on municipality-scale land-cover change in Mexico. Regional Environmental Change: 1–15.

[58] Cardenas F (2000) Consolidación y fortalecimiento de los programas ambientales en la cuenca media del río Chicamocha (Boyacá-Colombia). In: Cardenas F, editor. Desarrollo sostenible en los Andes de Colombia Provincias del Norte, Gutiérrez y Valderrama-Boyacá, Colombia. Bogotá, DC, Colombia: IDEADE, Pontificia Universidad Javeriana. 127–144.

[59] Rodríguez M (2008) Gobernabilidad, instituciones y medio ambiente en Colombia. Bogotá, D.C, Colombia: Editorial Gente Nueva.

[60] Sáenz H 2011 Aug 13 Mondoñedo: de desierto a bosque frondoso. Agencia de noticias Universidad Nacional de Colombia Bogotá, D.C, Colombia Available: http://www.unperiodico.unal.edu.co/dper/article/un-periodico-impreso-no-147/index.html. Accessed 2012 Feb 20.

[61] Meyerson FAB, Merino L, Durand J (2007) Migration and environment in the context of globalization. Frontiers in Ecology and the Environment 5: 182–190.

[62] Aide TM, Grau HR (2004) Globalization, Migration, and Latin American Ecosystems. Science 305: 1915–1916. 15448256 10.1126/science.1103179

[63] Pacheco P, Aguilar-Støen M, Börner J, Etter A, Putzel L, et al (2011) Landscape Transformation in Tropical Latin America: Assessing Trends and Policy Implications for REDD+. Forests 2: 1–29.

[64] Romero-Ruiz MH, Flantua SGA, Tansey K, Berrio JC (2011) Landscape transformations in savannas of northern South America: Land use/cover changes since 1987 in the Llanos Orientales of Colombia. Applied Geography 32: 766–776.

[65] DeFries R, Rudel TK, Uriarte M, Hansen M (2010) Deforestation driven by urban population growth and agricultural trade in the twenty-first century. Nature Geoscience 3: 178–181.

[66] Rudel TK, Schneider L, Uriarte M, Turner BL, DeFries R, et al (2009) Agricultural intensification and changes in cultivated areas, 1970–2005. Proceedings of the National Academy of Sciences of the United States of America 106: 20675–20680. 19955435 10.1073/pnas.0812540106 PMC2791618

[67] Etter A, Romero M, Sarmiento A (2011) Land use change (1970–2007) and the Carbon emissions in the Colombian Llanos. In: Hill M, Hanan NP, editors. Ecosystem Function in Savannas: measurement and modeling at landscape to global scales. Boca Raton, Florida: Taylor & Francis CRC Press. 383–402.

[68] Espinal CF, Martínez H, Pinzón N, Barrios C (2005a) La cadena de algodón en Colombia, una mirada global de su estructura y dinámica 1991–2005. Bogotá, DC, Colombia: Ministerio de Agricultura y Desarrollo Rural, Observatorio Agrocadenas Colombia. 44 p.

[69] Briggs JM, Schaafsma H, Trenkov D (2007) Woody vegetation expansion in a desert grassland: Prehistoric human impact? Journal of Arid Environments 69: 458–472.

[70] IDEAM (2010b) Informe anual sobre el estado del medio ambiente y los recursos naturales renovables en Colombia - Bosques 2009. Bogotá, D.C, Colombia: Instituto de Hidrología, Meteorología y Estudios Ambientales. 179 p.

[71] UNODC (2008) Monitoreo de cultivos de coca 2008. Bogotá, D.C, Colombia: United Nations Office for Drug and Crime. 107 p.

[72] Espinal CF, Martínez H, Pinzón N, Barrios C (2005b) La cadena de la papa en Colombia, una mirada global de su estructura y dinámica 1991–2005. Bogotá, DC, Colombia: Ministerio de Agricultura y Desarrollo Rural, Observatorio Agrocadenas Colombia. 28 p.

[73] Bruner AG, Gullison RE, Rice RE, da Fonseca GAB (2001) Effectiveness of parks in protecting tropical biodiversity. Science 291: 125–128. 11141563 10.1126/science.291.5501.125

[74] Forero-Medina G, Joppa L (2010) Representation of global and national conservation priorities by Colombia’s protected area network. PLoS ONE 5(10): e13210. 20967270 10.1371/journal.pone.0013210 PMC2953503

[75] Olson DM, Dinerstein E (2002) The Global 200: Priority ecoregions for global conservation. Annals of the Missouri Botanical Garden 89: 199–224.

[76] Cardenas D, Salinas N (2006) Especies maderables amenazadas: I parte. Serie Libro Rojo de plantas de Colombia. Bogotá, DC, Colombia: Instituto Amazónico de Investigaciones Científicas SINCHI. 169 p.

[77] Chazdon RL (2003) Tropical forest recovery: legacies of human impact and natural disturbances. Perspectives in Plant Ecology Evolution and Systematics 6: 51–71.

