# Supplementary figures and images for: Land Cover Change in Colombia: Surprising Forest Recovery Trends between 2001 and 2010
Source: PLoS One. 2012 Aug 29;7(8):e43943. doi: 10.1371/journal.pone.0043943 (PMC3430633; doi:10.1371/journal.pone.0043943)

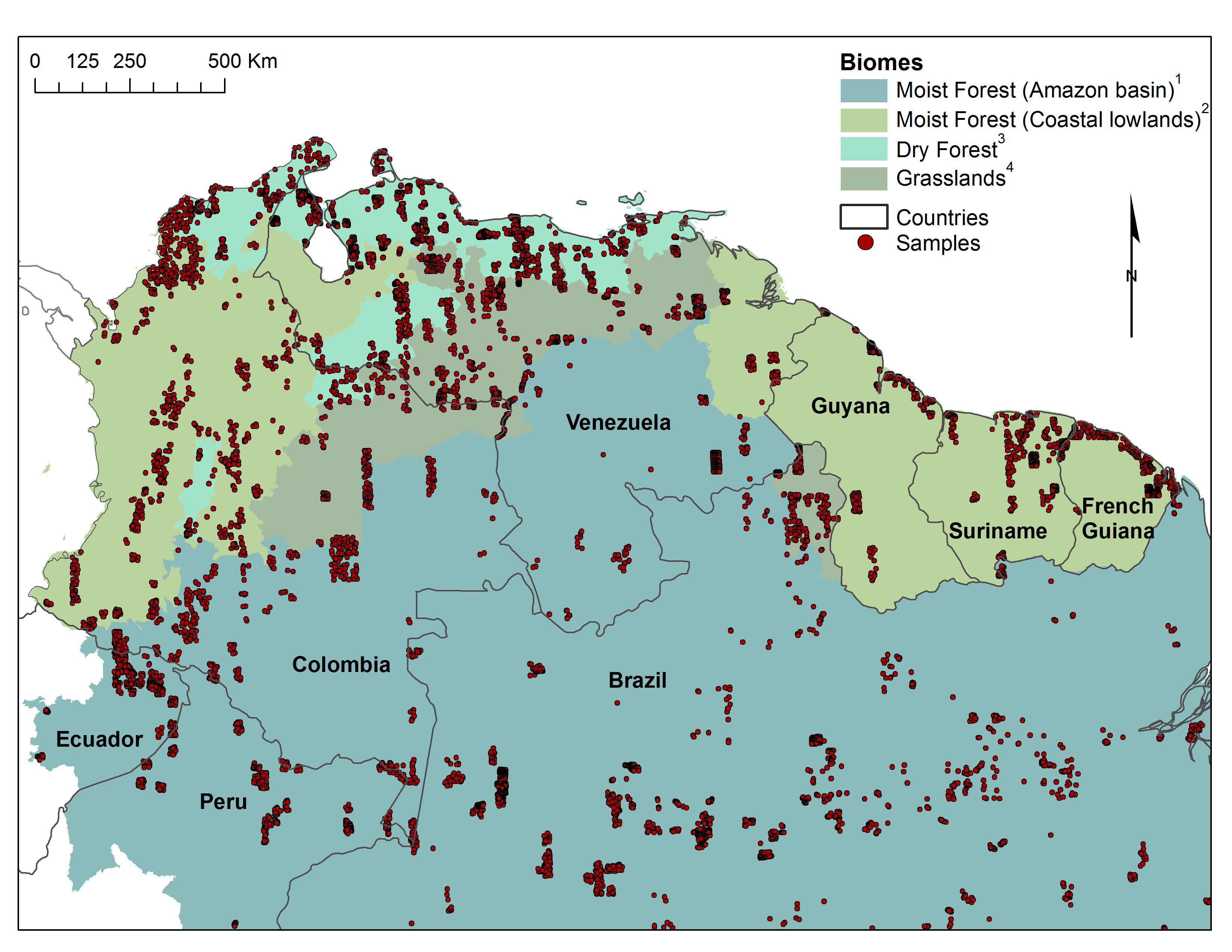

Supplement: Figure S1 — Distribution of reference data points collected from Google Earth within each of the three biomes which covered Colombia and neighboring countries. Biome description: 1. Tropical and Subtropical Moist Broadleaf Forest (Amazon basin section) 2. Tropical and Subtropical Moist Broadleaf Forest (Coastal lowlands section) 3. Tropical and Subtropical Dry Broadleaf Forest 4. Tropical and Subtropical Grasslands, Savannas and Shrublands (TIF) [file pone.0043943.s001.tif]
