# Supplementary material for: Land Cover Change in Colombia: Surprising Forest Recovery Trends between 2001 and 2010
Source: PLoS One. 2012 Aug 29;7(8):e43943. doi: 10.1371/journal.pone.0043943 (PMC3430633; doi:10.1371/journal.pone.0043943)
Supplement: Table S1 — Major biomes and ecoregions in Colombia. The names and area of the 6 major biomes and the 25 ecoregions in Colombia according to Olson et al. (2001). Note that the original 25 ecoregions were grouped into 13 ecoregions because some ecoregions only include one or a few municipalities. The name of the largest ecoregion was used as the name of the aggregation. (DOCX) [file pone.0043943.s002.docx]

## Supporting Information

**Table S1. Major biomes and ecoregions in Colombia.**

| **Biome names** | | | | | | | |
| --- | --- | --- | --- | --- | --- | --- | --- |
| **Olson et al.** | | | **This study** | | **Area (km^2^)** | | |
| Tropical and Subtropical Moist Broadleaf Forests | | | Moist Forest | | 906,096 | | |
| Tropical and Subtropical Dry Broadleaf Forests | | | Dry Forest | | 72,501 | | |
| Tropical and Subtropical Grasslands, Savannas,  Shrublands | | | Grasslands | | 134,447 | | |
| Montane Grasslands and Shrublands | | | Montane Grasslands | | 5,798 | | |
| Deserts and Xeric Shrublands | | | Deserts | | 25,788 | | |
| Mangroves | | | Mangroves | | 1,983 | | |
| **Ecoregion names** | | | | | | | |
| 1. Caquetá Moist Forest | | 1. Caquetá Moist | | | | 472,066 | |
| 2. Japurá-Solimoes-Negro Moist Forests | | |  | | |  | |
| 3. Napo Moist Forests | | |  | | |  | |
| 4. Negro-Branco Moist Forests | | |  | | |  | |
| 5. Rio Negro campinarana | | |  | | |  | |
| 6. Solimões-Japurá Moist Forest | | |  | | |  | |
| 7. Magdalena-Urabá Moist Forests | | 2. Mag-Urabá Moist | | | | 83,551 | |
| 8. Catatumbo Moist Forest | |  | | | |  | |
| 9. Chocó-Darién Moist Forests | | 3. Chocó-Darién Moist | | | | 74,353 | |
| 10. Western Ecuador Moist Forests | |  | | | |  | |
| 11. Cauca Valley Montane Forests | | 4. Cauca-Valley Montane | | | | 36,107 | |
| 12. Northwestern Andean Montane Forest | | 5. Northwestern Andean | | | | 49,185 | |
| 13. Cordillera Oriental Montane Forests | | 6. Cordillera Oriental | | | | 63,245 | |
| 14. Santa Marta Montane Forests | |  | | | |  | |
| 15. Northern Andean Montane Forests | | 7. Northern Andean | | | | 124,150 | |
| 16. Eastern Cordillera real Montane Forests | |  | | | |  | |
| 17. Northern Andean páramo | | 8. Northern Páramo | | | | 7,945 | |
| 18. Apure-Villavicencio dry Forests | | 9. Apure-Villavicencio | | | | 22,575 | |
| 19. Magdalena Valley dry Forests | | 10. Magdalena Valley Dry | | | | 22,436 | |
| 20. Cauca Valley Dry Forests | | |  | | |  | |
| 21. Patía Valley Dry Forests | | |  | | |  | |
| 22. Sinú Valley Dry Forests | | 11. Sinú-Valley Dry | | | | 28,782 | |
| 23. Guajira-Barranquilla Xeric Scrub | | 12. Guajira Xeric | | | | 27,770 | |
| 24. Alvarado Mangroves | |  | | | |  | |
| 25. Llanos | | 13. Llanos | | | | 134,447 | |

The names and area of the 6 major biomes and the 25 ecoregions in Colombia according to Olson et al. (2001). Note that the original 25 ecoregions were grouped into 13 ecoregions because some ecoregions only include one or a few municipalities. The name of the largest ecoregion was used as the name of the aggregation.
