# Supplementary material for: Land Cover Change in Colombia: Surprising Forest Recovery Trends between 2001 and 2010
Source: PLoS One. 2012 Aug 29;7(8):e43943. doi: 10.1371/journal.pone.0043943 (PMC3430633; doi:10.1371/journal.pone.0043943)
Supplement: Table S2 — Woody vegetation net gain and loss for all municipalities in Colombia. Thirty three municipalities were not included because they did not have any woody vegetation. (DOCX) [file pone.0043943.s003.docx]

## Supporting Information

**Table S2. Woody vegetation net gain and loss for all municipalities in Colombia.**

| **Department** | **Municipality** | **Ecoregion** | **Area (km²)** | **Net Change (km²)** | **Woody (R)** | ***p*-value** |
| --- | --- | --- | --- | --- | --- | --- |
| **Amazonas** | El Encanto | Caquetá Moist | 11,358 | 58.9 | 0.30 | 0.393 |
|  | La Chorrera | Caquetá Moist | 12,496 | 104.4 | 0.38 | 0.274 |
|  | La Pedrera | Caquetá Moist | 13,816 | -45.4 | -0.10 | 0.785 |
|  | La Victoria | Caquetá Moist | 1,410 | 3.5 | 0.35 | 0.314 |
|  | Leticia | Caquetá Moist | 6,168 | -113.8 | -0.61 | 0.062 |
|  | Mirití Paraná | Caquetá Moist | 17,118 | -43.1 | -0.12 | 0.734 |
|  | Puerto Alegría | Caquetá Moist | 8,768 | -39.4 | -0.30 | 0.392 |
|  | Puerto Arica | Caquetá Moist | 13,913 | 81.2 | 0.12 | 0.743 |
|  | Puerto Nariño | Caquetá Moist | 1,527 | -52.0 | -0.59 | 0.073 |
|  | Puerto Santander | Caquetá Moist | 15,051 | 106.4 | 0.28 | 0.437 |
|  | Tarapacá | Caquetá Moist | 9,121 | 12.0 | 0.03 | 0.937 |
| **Antioquia** | Abejorral | Cauca-Valley Montane | 494 | 99.3 | 0.76 | 0.011 |
|  | Abriaquí | Cauca-Valley Montane | 290 | 29.3 | 0.60 | 0.068 |
|  | Alejandría | Northern Andean | 136 | 12.0 | 0.58 | 0.079 |
|  | Amagá | Cauca-Valley Montane | 86 | 9.1 | 0.47 | 0.175 |
|  | Amalfi | Northern Andean | 1,198 | 9.4 | 0.07 | 0.857 |
|  | Andes | Cauca-Valley Montane | 439 | 14.8 | 0.20 | 0.603 |
|  | Angelópolis | Cauca-Valley Montane | 87 | 9.8 | 0.48 | 0.157 |
|  | Angostura | Northern Andean | 414 | 10.0 | 0.24 | 0.500 |
|  | Anorí | Northern Andean | 1,422 | 27.1 | 0.19 | 0.600 |
|  | Anza | Cauca-Valley Montane | 254 | 16.6 | 0.42 | 0.222 |
|  | Apartadó | Northwestern Andean | 611 | 6.7 | 0.03 | 0.939 |
|  | Arboletes | Mag-Urabá Moist | 753 | -8.6 | -0.22 | 0.548 |
|  | Argelia | Northern Andean | 262 | 4.1 | 0.13 | 0.714 |
|  | Armenia | Magdalena Valley Dry | 113 | 20.7 | 0.77 | 0.009 |
|  | Barbosa | Northern Andean | 228 | 22.4 | 0.68 | 0.029 |
|  | Bello | Northern Andean | 144 | 1.8 | 0.22 | 0.535 |
|  | Belmira | Northern Andean | 297 | -2.5 | -0.08 | 0.824 |
|  | Betania | Cauca-Valley Montane | 197 | 6.6 | 0.18 | 0.670 |
|  | Betulia | Cauca-Valley Montane | 279 | 50.0 | 0.72 | 0.019 |
|  | Briceño | Cauca-Valley Montane | 349 | -11.7 | -0.24 | 0.503 |
|  | Buriticá | Cauca-Valley Montane | 360 | 6.3 | 0.20 | 0.578 |
|  | Cáceres | Mag-Urabá Moist | 1,913 | 9.5 | 0.08 | 0.832 |
|  | Caicedo | Cauca-Valley Montane | 237 | 3.2 | 0.09 | 0.808 |
|  | Caldas | Northern Andean | 162 | 9.9 | 0.33 | 0.349 |
|  | Campamento | Northern Andean | 222 | 38.9 | 0.64 | 0.046 |
|  | Cañasgordas | Cauca-Valley Montane | 388 | 79.8 | 0.67 | 0.035 |
|  | Caracolí | Northern Andean | 265 | 21.9 | 0.48 | 0.163 |
|  | Caramanta | Cauca-Valley Montane | 95 | 4.2 | 0.41 | 0.241 |
|  | Carepa | Chocó-Darién Moist | 354 | -1.3 | -0.02 | 0.960 |
|  | Carolina | Northern Andean | 161 | 7.1 | 0.43 | 0.216 |
|  | Caucasia | Mag-Urabá Moist | 1,383 | 28.1 | 0.50 | 0.142 |
|  | Chigorodó | Chocó-Darién Moist | 703 | 32.8 | 0.43 | 0.213 |
|  | Cisneros | Northern Andean | 38 | 0.0 | 0.00 | 0.995 |
|  | Ciudad Bolívar | Cauca-Valley Montane | 344 | -5.9 | -0.05 | 0.907 |
|  | Cocorná | Northern Andean | 210 | -3.9 | -0.12 | 0.741 |
|  | Concepción | Northern Andean | 159 | 8.5 | 0.37 | 0.298 |
|  | Concordia | Cauca-Valley Montane | 241 | 18.7 | 0.57 | 0.089 |
|  | Copacabana | Northern Andean | 55 | 3.1 | 0.45 | 0.191 |
|  | Dabeiba | Northwestern Andean | 1,811 | 136.4 | 0.31 | 0.498 |
|  | Don Matías | Northern Andean | 150 | -0.6 | -0.05 | 0.890 |
|  | Ebéjico | Cauca-Valley Montane | 237 | 70.7 | 0.77 | 0.009 |
|  | El Bagre | Mag-Urabá Moist | 1,634 | -12.6 | -0.10 | 0.784 |
|  | El Carmen de Viboral | Northern Andean | 461 | -7.1 | -0.18 | 0.613 |
|  | El Santuario | Northern Andean | 67 | 2.8 | 0.49 | 0.147 |
|  | Entrerrios | Northern Andean | 198 | 0.2 | 0.04 | 0.919 |
|  | Envigado | Northern Andean | 43 | -0.5 | -0.09 | 0.808 |
|  | Fredonia | Cauca-Valley Montane | 249 | 25.6 | 0.80 | 0.006 |
|  | Frontino | Northwestern Andean | 1,338 | 20.4 | 0.05 | 0.891 |
|  | Giraldo | Cauca-Valley Montane | 135 | 10.0 | 0.56 | 0.093 |
|  | Girardota | Northern Andean | 86 | 4.8 | 0.61 | 0.060 |
|  | Gómez Plata | Northern Andean | 364 | 31.6 | 0.64 | 0.046 |
|  | Granada | Northern Andean | 209 | 23.0 | 0.62 | 0.058 |
|  | Guadalupe | Northern Andean | 73 | 5.1 | 0.42 | 0.225 |
|  | Guarne | Northern Andean | 167 | 7.2 | 0.40 | 0.247 |
|  | Guatapé | Northern Andean | 73 | -1.2 | -0.20 | 0.574 |
|  | Heliconia | Cauca-Valley Montane | 115 | 18.3 | 0.48 | 0.160 |
|  | Hispania | Magdalena Valley Dry | 56 | 7.3 | 0.48 | 0.159 |
|  | Itagui | Northern Andean | 18 | 0.7 | 0.25 | 0.478 |
|  | Ituango | Northwestern Andean | 2,229 | -69.9 | -0.19 | 0.596 |
|  | Jardín | Cauca-Valley Montane | 228 | 38.8 | 0.89 | 0.001 |
|  | Jericó | Cauca-Valley Montane | 202 | 16.6 | 0.74 | 0.015 |
|  | La Ceja | Cauca-Valley Montane | 131 | 10.4 | 0.66 | 0.037 |
|  | La Estrella | Northern Andean | 33 | 2.5 | 0.52 | 0.122 |
|  | La Pintada | Magdalena Valley Dry | 70 | 5.4 | 0.89 | 0.001 |
|  | La Unión | Cauca-Valley Montane | 202 | 25.3 | 0.70 | 0.025 |
|  | Liborina | Cauca-Valley Montane | 225 | 22.3 | 0.55 | 0.101 |
|  | Maceo | Northern Andean | 450 | -28.9 | -0.41 | 0.239 |
|  | Marinilla | Northern Andean | 108 | 0.5 | 0.39 | 0.264 |
|  | Medellín | Northern Andean | 375 | 35.9 | 0.62 | 0.055 |
|  | Montebello | Cauca-Valley Montane | 77 | 12.7 | 0.41 | 0.236 |
|  | Murindó | Chocó-Darién Moist | 1,358 | -92.9 | -0.22 | 0.632 |
|  | Mutatá | Chocó-Darién Moist | 1,261 | -67.8 | -0.43 | 0.247 |
|  | Nariño | Northern Andean | 328 | 42.0 | 0.67 | 0.050 |
|  | Nechí | Mag-Urabá Moist | 957 | 16.3 | 0.15 | 0.671 |
|  | Necoclí | Mag-Urabá Moist | 1,321 | -32.1 | -0.19 | 0.604 |
|  | Olaya | Cauca-Valley Montane | 96 | 19.5 | 0.57 | 0.089 |
|  | Peñol | Northern Andean | 161 | 3.3 | 0.39 | 0.259 |
|  | Pequé | Cauca-Valley Montane | 411 | 26.0 | 0.51 | 0.133 |
|  | Pueblorrico | Cauca-Valley Montane | 76 | 8.8 | 0.34 | 0.332 |
|  | Puerto Berrío | Northern Andean | 1,190 | -20.7 | -0.22 | 0.546 |
|  | Puerto Nare | Northern Andean | 621 | 13.7 | 0.27 | 0.449 |
|  | Puerto Triunfo | Mag-Urabá Moist | 369 | -6.7 | -0.33 | 0.353 |
|  | Remedios | Northern Andean | 2,045 | -384.5 | -0.87 | 0.001 |
|  | Retiro | Northern Andean | 264 | -14.6 | -0.45 | 0.189 |
|  | Rionegro | Northern Andean | 219 | -2.5 | -0.25 | 0.478 |
|  | Sabanalarga | Cauca-Valley Montane | 257 | 21.9 | 0.65 | 0.040 |
|  | Sabaneta | Northern Andean | 18 | 0.9 | 0.39 | 0.269 |
|  | Salgar | Cauca-Valley Montane | 435 | 65.5 | 0.67 | 0.036 |
|  | San Andrés de Cuerquía | Cauca-Valley Montane | 200 | 16.0 | 0.50 | 0.145 |
|  | San Carlos | Northern Andean | 744 | 13.4 | 0.12 | 0.750 |
|  | San Francisco | Northern Andean | 383 | 4.1 | 0.06 | 0.875 |
|  | San Jerónimo | Cauca-Valley Montane | 138 | 55.8 | 0.87 | 0.001 |
|  | San José de la Montaña | Cauca-Valley Montane | 154 | 10.2 | 0.42 | 0.224 |
|  | San Juan de Urabá | Mag-Urabá Moist | 237 | -5.4 | -0.41 | 0.239 |
|  | San Luis | Northern Andean | 450 | -54.5 | -0.76 | 0.011 |
|  | San Pedro | Northern Andean | 224 | 0.3 | 0.04 | 0.919 |
|  | San Pedro de Urabá | Mag-Urabá Moist | 620 | 13.3 | 0.33 | 0.360 |
|  | San Rafael | Northern Andean | 345 | 20.2 | 0.35 | 0.319 |
|  | San Roque | Northern Andean | 439 | 30.2 | 0.45 | 0.193 |
|  | San Vicente | Northern Andean | 213 | 7.6 | 0.47 | 0.169 |
|  | Santa Bárbara | Cauca-Valley Montane | 195 | 28.2 | 0.63 | 0.050 |
|  | Santa Rosa de Osos | Northern Andean | 876 | 44.0 | 0.78 | 0.008 |
|  | Santafé de Antioquia | Cauca-Valley Montane | 464 | 24.1 | 0.51 | 0.128 |
|  | Santo Domingo | Northern Andean | 281 | 8.7 | 0.27 | 0.458 |
|  | Segovia | Northern Andean | 1,154 | -103.4 | -0.83 | 0.003 |
|  | Sonson | Northern Andean | 1,315 | 12.5 | 0.16 | 0.657 |
|  | Sopetrán | Cauca-Valley Montane | 213 | 41.0 | 0.75 | 0.012 |
|  | Támesis | Cauca-Valley Montane | 231 | 11.0 | 0.80 | 0.005 |
|  | Taraza | Cauca-Valley Montane | 1,582 | 39.8 | 0.21 | 0.565 |
|  | Tarso | Cauca-Valley Montane | 123 | 21.4 | 0.72 | 0.018 |
|  | Titiribí | Cauca-Valley Montane | 136 | 39.1 | 0.81 | 0.005 |
|  | Toledo | Cauca-Valley Montane | 134 | 21.9 | 0.82 | 0.003 |
|  | Turbo | Mag-Urabá Moist | 2,994 | 52.0 | 0.10 | 0.790 |
|  | Uramita | Northwestern Andean | 272 | 26.8 | 0.40 | 0.248 |
|  | Urrao | Northwestern Andean | 2,742 | 247.3 | 0.38 | 0.534 |
|  | Valdivia | Cauca-Valley Montane | 600 | -0.2 | 0.00 | 0.998 |
|  | Valparaiso | Magdalena Valley Dry | 125 | 3.1 | 0.27 | 0.459 |
|  | Vegachí | Northern Andean | 531 | -51.9 | -0.45 | 0.191 |
|  | Venecia | Magdalena Valley Dry | 145 | 35.9 | 0.79 | 0.006 |
|  | Vigía del Fuerte | Chocó-Darién Moist | 1,617 | -106.5 | -0.34 | 0.372 |
|  | Yalí | Northern Andean | 484 | -31.4 | -0.39 | 0.271 |
|  | Yarumal | Northern Andean | 687 | 21.8 | 0.34 | 0.340 |
|  | Yolombó | Northern Andean | 941 | 27.0 | 0.29 | 0.412 |
|  | Yondó | Mag-Urabá Moist | 1,967 | -144.6 | -0.34 | 0.337 |
|  | Zaragoza | Mag-Urabá Moist | 1,157 | -38.7 | -0.43 | 0.210 |
| **Arauca** | Arauca | Llanos | 5,525 | 16.9 | 0.04 | 0.905 |
|  | Arauquita | Apure-Villavicencio | 3,218 | -470.9 | -0.63 | 0.049 |
|  | Cravo Norte | Llanos | 5,538 | -25.8 | -0.27 | 0.450 |
|  | Fortul | Cordillera Oriental | 1,067 | -126.3 | -0.85 | 0.032 |
|  | Puerto Rondón | Apure-Villavicencio | 2,251 | -72.8 | -0.36 | 0.303 |
|  | Saravena | Apure-Villavicencio | 881 | -70.0 | -0.48 | 0.156 |
|  | Tame | Apure-Villavicencio | 5,433 | -408.7 | -0.74 | 0.014 |
| **Atlántico** | Baranoa | Guajira Xeric | 121 | 0.1 | 0.43 | 0.210 |
|  | Barranquilla | Guajira Xeric | 149 | 0.6 | 0.45 | 0.191 |
|  | Campo de la Cruz | Sinú-Valley Dry | 106 | 13.9 | 0.56 | 0.092 |
|  | Candelaria | Sinú-Valley Dry | 136 | 10.3 | 0.47 | 0.174 |
|  | Galapa | Guajira Xeric | 99 | 0.0 | 0.19 | 0.598 |
|  | Juan de Acosta | Guajira Xeric | 177 | 1.8 | 0.47 | 0.166 |
|  | Luruaco | Guajira Xeric | 251 | 7.6 | 0.64 | 0.046 |
|  | Malambó | Guajira Xeric | 102 | 0.7 | 0.56 | 0.090 |
|  | Manatí | Guajira Xeric | 212 | 2.7 | 0.66 | 0.039 |
|  | Palmar de Varela | Guajira Xeric | 92 | 0.1 | 0.16 | 0.664 |
|  | Piojó | Guajira Xeric | 249 | 5.1 | 0.63 | 0.049 |
|  | Ponedera | Guajira Xeric | 204 | -0.4 | -0.17 | 0.635 |
|  | Puerto Colombia | Guajira Xeric | 76 | 1.4 | 0.51 | 0.202 |
|  | Repelón | Guajira Xeric | 340 | 7.3 | 0.54 | 0.105 |
|  | Sabanagrande | Guajira Xeric | 42 | 0.2 | 0.30 | 0.404 |
|  | Sabanalarga | Guajira Xeric | 403 | 0.5 | 0.69 | 0.026 |
|  | Santa Lucía | Sinú-Valley Dry | 55 | 5.4 | 0.38 | 0.275 |
|  | Santo Tomás | Guajira Xeric | 66 | -0.3 | -0.23 | 0.528 |
|  | Soledad | Guajira Xeric | 61 | 0.9 | 0.35 | 0.319 |
|  | Suan | Sinú-Valley Dry | 44 | 7.4 | 0.56 | 0.094 |
|  | Tubará | Guajira Xeric | 172 | 6.1 | 0.45 | 0.195 |
|  | Usiacurí | Guajira Xeric | 103 | 0.7 | 0.63 | 0.053 |
| **Bogotá  D.C** | Santa Fe de Bogotá | Northern Andean | 1,654 | 13.1 | 0.52 | 0.126 |
| **Bolívar** | Achí | Mag-Urabá Moist | 973 | 120.9 | 0.58 | 0.078 |
|  | Altos del Rosario | Mag-Urabá Moist | 278 | 29.8 | 0.60 | 0.068 |
|  | Arenal | Northern Andean | 480 | 8.0 | 0.13 | 0.714 |
|  | Arjona | Guajira Xeric | 605 | 13.3 | 0.38 | 0.284 |
|  | Arroyohondo | Sinú-Valley Dry | 163 | 18.8 | 0.45 | 0.192 |
|  | Barranco de Loba | Mag-Urabá Moist | 442 | 68.0 | 0.73 | 0.016 |
|  | Calamar | Sinú-Valley Dry | 268 | 35.8 | 0.45 | 0.188 |
|  | Cantagallo | Northern Andean | 882 | -27.2 | -0.19 | 0.604 |
|  | Cartagena | Guajira Xeric | 635 | 25.1 | 0.59 | 0.120 |
|  | Cicuco | Mag-Urabá Moist | 128 | -2.1 | -0.23 | 0.526 |
|  | Clemencia | Guajira Xeric | 78 | 0.4 | 0.51 | 0.133 |
|  | Córdoba | Mag-Urabá Moist | 603 | 35.6 | 0.32 | 0.370 |
|  | El Carmen de Bolívar | Mag-Urabá Moist | 929 | 147.6 | 0.54 | 0.110 |
|  | El Guamo | Mag-Urabá Moist | 411 | 17.5 | 0.65 | 0.044 |
|  | El Peñón | Mag-Urabá Moist | 303 | 71.9 | 0.74 | 0.015 |
|  | Hatillo de Loba | Mag-Urabá Moist | 218 | 18.3 | 0.48 | 0.164 |
|  | Magangué | Mag-Urabá Moist | 1,158 | -1.6 | -0.03 | 0.937 |
|  | Mahates | Guajira Xeric | 431 | 4.3 | 0.28 | 0.428 |
|  | Margarita | Mag-Urabá Moist | 288 | 15.1 | 0.47 | 0.167 |
|  | Maria la Baja | Guajira Xeric | 536 | 8.5 | 0.19 | 0.604 |
|  | Mompós | Mag-Urabá Moist | 667 | 30.4 | 0.54 | 0.110 |
|  | Montecristo | Northern Andean | 2,095 | -12.3 | -0.09 | 0.802 |
|  | Morales | Northern Andean | 1,344 | 49.7 | 0.37 | 0.286 |
|  | Pinillos | Mag-Urabá Moist | 752 | 7.9 | 0.10 | 0.775 |
|  | Regidor | Mag-Urabá Moist | 184 | 33.9 | 0.67 | 0.033 |
|  | Rioviejo | Mag-Urabá Moist | 1,285 | 178.1 | 0.64 | 0.045 |
|  | San Cristóbal | Guajira Xeric | 39 | 0.0 | -0.04 | 0.915 |
|  | San Estanislao | Guajira Xeric | 213 | 2.2 | 0.26 | 0.467 |
|  | San Fernando | Mag-Urabá Moist | 326 | 37.4 | 0.69 | 0.027 |
|  | San Jacinto | Mag-Urabá Moist | 427 | 67.2 | 0.58 | 0.077 |
|  | San Jacinto del Cauca | Mag-Urabá Moist | 560 | 4.2 | 0.07 | 0.846 |
|  | San Juan Nepomuceno | Mag-Urabá Moist | 625 | 98.9 | 0.78 | 0.008 |
|  | San Martín de Loba | Mag-Urabá Moist | 463 | 60.2 | 0.55 | 0.096 |
|  | San Pablo | Northern Andean | 2,002 | -80.3 | -0.41 | 0.243 |
|  | Santa Catalina | Guajira Xeric | 169 | 2.6 | 0.66 | 0.076 |
|  | Santa Rosa | Guajira Xeric | 159 | 1.6 | 0.57 | 0.086 |
|  | Santa Rosa del Sur | Northern Andean | 2,411 | -54.2 | -0.50 | 0.143 |
|  | Simití | Mag-Urabá Moist | 1,350 | 27.5 | 0.28 | 0.438 |
|  | Soplaviento | Guajira Xeric | 96 | 0.1 | 0.03 | 0.943 |
|  | Talaigua Nuevo | Mag-Urabá Moist | 234 | 5.4 | 0.19 | 0.594 |
|  | Tiquisio | Mag-Urabá Moist | 774 | 134.4 | 0.62 | 0.055 |
|  | Turbaco | Guajira Xeric | 181 | 0.2 | 0.45 | 0.194 |
|  | Turbana | Guajira Xeric | 150 | 1.0 | 0.52 | 0.126 |
|  | Villanueva | Guajira Xeric | 141 | 0.2 | 0.21 | 0.559 |
|  | Zambrano | Mag-Urabá Moist | 324 | 23.3 | 0.49 | 0.151 |
| **Boyacá** | Almeida | Cordillera Oriental | 50 | 0.8 | 0.44 | 0.205 |
|  | Aquitania | Northern Páramo | 855 | 9.5 | 0.43 | 0.214 |
|  | Arcabuco | Northern Andean | 166 | 7.1 | 0.66 | 0.037 |
|  | Belén | Northern Andean | 178 | 4.8 | 0.56 | 0.091 |
|  | Berbeo | Cordillera Oriental | 63 | 0.6 | 0.12 | 0.776 |
|  | Betéitiva | Northern Andean | 103 | 0.1 | 0.45 | 0.196 |
|  | Boavita | Northern Andean | 150 | 2.2 | 0.69 | 0.028 |
|  | Briceño | Northern Andean | 45 | 9.3 | 0.56 | 0.094 |
|  | Buenavista | Northern Andean | 90 | 14.8 | 0.90 | 0.000 |
|  | Caldas | Northern Andean | 73 | 0.1 | 0.63 | 0.052 |
|  | Campohermoso | Cordillera Oriental | 294 | 1.2 | 0.05 | 0.882 |
|  | Cerinza | Northern Andean | 52 | 0.5 | 0.58 | 0.080 |
|  | Chinavita | Cordillera Oriental | 162 | 3.2 | 0.21 | 0.552 |
|  | Chiquinquirá | Northern Andean | 152 | 3.8 | 0.54 | 0.108 |
|  | Chíquiza | Northern Andean | 94 | 4.3 | 0.76 | 0.010 |
|  | Chiscas | Northern Páramo | 648 | -1.7 | -0.04 | 0.916 |
|  | Chita | Northern Páramo | 753 | -20.8 | -0.42 | 0.224 |
|  | Chitaraque | Northern Andean | 176 | 6.5 | 0.43 | 0.209 |
|  | Chivatá | Northern Andean | 51 | 0.0 | 0.29 | 0.416 |
|  | Chivor | Cordillera Oriental | 118 | 7.0 | 0.27 | 0.457 |
|  | Ciénega | Cordillera Oriental | 54 | 0.0 | -0.12 | 0.737 |
|  | Cómbita | Northern Andean | 163 | 1.5 | 0.45 | 0.188 |
|  | Coper | Northern Andean | 120 | 36.9 | 0.81 | 0.004 |
|  | Corrales | Northern Andean | 54 | 0.8 | 0.67 | 0.035 |
|  | Covarachía | Northern Andean | 99 | 1.9 | 0.68 | 0.030 |
|  | Cubará | Cordillera Oriental | 1,186 | 7.9 | 0.05 | 0.931 |
|  | Duitama | Northern Andean | 257 | 10.3 | 0.53 | 0.113 |
|  | El Cocuy | Northern Páramo | 225 | 1.2 | 0.51 | 0.133 |
|  | El Espino | Northern Andean | 73 | 3.0 | 0.62 | 0.056 |
|  | Floresta | Northern Andean | 105 | 2.2 | 0.84 | 0.003 |
|  | Gachantivá | Northern Andean | 84 | 3.4 | 0.87 | 0.001 |
|  | Gámeza | Northern Páramo | 117 | 0.9 | 0.40 | 0.250 |
|  | Garagoa | Cordillera Oriental | 181 | -6.4 | -0.31 | 0.385 |
|  | Guacamayas | Northern Andean | 63 | 3.8 | 0.62 | 0.058 |
|  | Guateque | Cordillera Oriental | 35 | 0.0 | -0.17 | 0.631 |
|  | Guayata | Cordillera Oriental | 83 | 0.6 | 0.10 | 0.785 |
|  | Guicán | Northern Páramo | 953 | 21.8 | 0.50 | 0.173 |
|  | Jenesano | Cordillera Oriental | 53 | 0.0 | -0.06 | 0.873 |
|  | La Capilla | Cordillera Oriental | 56 | -1.6 | -0.30 | 0.431 |
|  | La Uvita | Northern Páramo | 171 | 5.2 | 0.34 | 0.342 |
|  | La Victoria | Northern Andean | 33 | 0.8 | 0.07 | 0.849 |
|  | Labranzagrande | Cordillera Oriental | 624 | -6.5 | -0.12 | 0.743 |
|  | Macanal | Cordillera Oriental | 201 | 11.2 | 0.51 | 0.128 |
|  | Maripí | Northern Andean | 164 | 50.9 | 0.84 | 0.005 |
|  | Miraflores | Cordillera Oriental | 261 | 10.2 | 0.35 | 0.324 |
|  | Mongua | Northern Páramo | 357 | 11.6 | 0.36 | 0.309 |
|  | Mongui | Northern Páramo | 58 | 0.8 | 0.84 | 0.003 |
|  | Moniquirá | Northern Andean | 222 | 26.9 | 0.80 | 0.005 |
|  | Muzo | Northern Andean | 135 | 24.3 | 0.70 | 0.024 |
|  | Nobsa | Northern Andean | 50 | 0.9 | 0.71 | 0.022 |
|  | Nuevo Colón | Northern Andean | 54 | 0.0 | 0.52 | 0.126 |
|  | Oicatá | Northern Andean | 49 | 0.0 | 0.35 | 0.324 |
|  | Otanche | Northern Andean | 486 | 53.4 | 0.60 | 0.067 |
|  | Pachavita | Cordillera Oriental | 72 | -0.3 | -0.10 | 0.791 |
|  | Páez | Cordillera Oriental | 356 | 14.2 | 0.29 | 0.423 |
|  | Paipa | Northern Andean | 402 | 13.9 | 0.51 | 0.134 |
|  | Pajarito | Cordillera Oriental | 309 | 20.6 | 0.47 | 0.285 |
|  | Panqueba | Northern Andean | 44 | 0.5 | 0.43 | 0.214 |
|  | Pauna | Northern Andean | 303 | 60.0 | 0.66 | 0.052 |
|  | Paya | Cordillera Oriental | 433 | -6.7 | -0.11 | 0.755 |
|  | Paz de Río | Northern Andean | 126 | 0.8 | 0.64 | 0.046 |
|  | Pesca | Northern Páramo | 294 | -7.2 | -0.39 | 0.271 |
|  | Pisba | Cordillera Oriental | 431 | -9.7 | -0.16 | 0.668 |
|  | Puerto Boyacá | Mag-Urabá Moist | 1,536 | 35.4 | 0.62 | 0.057 |
|  | Quípama | Northern Andean | 177 | 29.6 | 0.55 | 0.097 |
|  | Ramiriquí | Cordillera Oriental | 121 | -1.8 | -0.23 | 0.522 |
|  | Ráquira | Northern Andean | 209 | 6.0 | 0.54 | 0.109 |
|  | Rondón | Cordillera Oriental | 153 | -1.3 | -0.08 | 0.831 |
|  | Saboyá | Northern Andean | 255 | 3.3 | 0.68 | 0.031 |
|  | Samacá | Northern Andean | 170 | 3.0 | 0.65 | 0.041 |
|  | San Eduardo | Cordillera Oriental | 100 | 1.8 | 0.16 | 0.756 |
|  | San José de Pare | Northern Andean | 78 | 1.4 | 0.37 | 0.299 |
|  | San Luis de Gaceno | Cordillera Oriental | 475 | 7.8 | 0.24 | 0.513 |
|  | San Mateo | Northern Andean | 114 | 9.9 | 0.52 | 0.120 |
|  | San Miguel de Sema | Northern Andean | 82 | 1.8 | 0.36 | 0.300 |
|  | San Pablo de Borbur | Northern Andean | 171 | 34.3 | 0.78 | 0.008 |
|  | Santa María | Cordillera Oriental | 305 | 14.9 | 0.28 | 0.431 |
|  | Santa Rosa de Viterbo | Northern Andean | 85 | 2.2 | 0.49 | 0.148 |
|  | Santana | Northern Andean | 68 | 0.7 | 0.52 | 0.121 |
|  | Sativanorte | Northern Andean | 151 | 0.4 | 0.57 | 0.085 |
|  | Siachoque | Northern Páramo | 176 | -4.0 | -0.35 | 0.354 |
|  | Soatá | Northern Andean | 127 | 0.9 | 0.18 | 0.627 |
|  | Socha | Northern Páramo | 164 | 1.0 | 0.85 | 0.002 |
|  | Socotá | Northern Páramo | 619 | -4.4 | -0.08 | 0.820 |
|  | Sogamoso | Northern Páramo | 200 | 0.7 | 0.84 | 0.002 |
|  | Somondoco | Cordillera Oriental | 74 | -3.8 | -0.28 | 0.427 |
|  | Sotaquirá | Northern Andean | 261 | 9.9 | 0.32 | 0.366 |
|  | Susacón | Northern Andean | 195 | 2.1 | 0.41 | 0.242 |
|  | Tasco | Northern Páramo | 228 | 0.0 | 0.14 | 0.705 |
|  | Tenza | Cordillera Oriental | 44 | 0.0 | 0.29 | 0.416 |
|  | Tibaná | Cordillera Oriental | 94 | -0.1 | -0.02 | 0.950 |
|  | Tinjacá | Northern Andean | 77 | 0.6 | 0.47 | 0.175 |
|  | Tipacoque | Northern Andean | 71 | 1.3 | 0.53 | 0.114 |
|  | Toguí | Northern Andean | 88 | 6.4 | 0.80 | 0.006 |
|  | Tota | Northern Páramo | 180 | -0.2 | -0.25 | 0.488 |
|  | Tunja | Northern Andean | 142 | 0.1 | 0.24 | 0.496 |
|  | Tunungua | Northern Andean | 40 | 8.2 | 0.38 | 0.285 |
|  | Turmequé | Northern Andean | 86 | 0.0 | 0.41 | 0.244 |
|  | Tutazá | Northern Páramo | 118 | 0.1 | 0.41 | 0.236 |
|  | Umbita | Cordillera Oriental | 156 | -0.8 | -0.28 | 0.441 |
|  | Ventaquemada | Northern Andean | 135 | 0.0 | 0.30 | 0.401 |
|  | Villa de Leyva | Northern Andean | 112 | 4.0 | 0.93 | 0.000 |
|  | Viracacha | Cordillera Oriental | 57 | 0.0 | -0.09 | 0.811 |
|  | Zetaquirá | Cordillera Oriental | 292 | -1.1 | -0.05 | 0.899 |
| **Caldas** | Aguadas | Cauca-Valley Montane | 479 | 69.2 | 0.78 | 0.008 |
|  | Anserma | Cauca-Valley Montane | 213 | 20.3 | 0.58 | 0.077 |
|  | Aranzazu | Cauca-Valley Montane | 147 | 6.4 | 0.16 | 0.655 |
|  | Belalcázar | Cauca-Valley Montane | 112 | 14.9 | 0.48 | 0.162 |
|  | Chinchiná | Cauca-Valley Montane | 115 | 7.3 | 0.29 | 0.417 |
|  | Filadelfia | Cauca-Valley Montane | 205 | 18.1 | 0.57 | 0.088 |
|  | La Dorada | Mag-Urabá Moist | 564 | 11.6 | 0.47 | 0.166 |
|  | La Merced | Cauca-Valley Montane | 91 | 6.4 | 0.53 | 0.113 |
|  | Manizales | Cauca-Valley Montane | 441 | 30.0 | 0.43 | 0.209 |
|  | Manzanares | Northern Andean | 183 | -13.3 | -0.45 | 0.195 |
|  | Marmato | Magdalena Valley Dry | 40 | -1.1 | -0.19 | 0.603 |
|  | Marquetalia | Northern Andean | 97 | -19.9 | -0.68 | 0.030 |
|  | Marulanda | Northern Andean | 370 | -6.8 | -0.15 | 0.677 |
|  | Neira | Cauca-Valley Montane | 366 | 15.4 | 0.25 | 0.482 |
|  | Norcasia | Northern Andean | 227 | 0.9 | 0.03 | 0.944 |
|  | Pácora | Cauca-Valley Montane | 261 | 42.5 | 0.65 | 0.043 |
|  | Palestina | Cauca-Valley Montane | 112 | 2.1 | 0.11 | 0.772 |
|  | Pensilvania | Northern Andean | 518 | -61.8 | -0.65 | 0.082 |
|  | Riosucio | Cauca-Valley Montane | 386 | 24.2 | 0.47 | 0.166 |
|  | Risaralda | Cauca-Valley Montane | 90 | 4.7 | 0.42 | 0.225 |
|  | Salamina | Cauca-Valley Montane | 390 | 23.0 | 0.42 | 0.232 |
|  | Samaná | Northern Andean | 779 | -32.8 | -0.33 | 0.350 |
|  | San José | Cauca-Valley Montane | 62 | 5.8 | 0.33 | 0.359 |
|  | Supía | Cauca-Valley Montane | 123 | 5.8 | 0.37 | 0.298 |
|  | Victoria | Northern Andean | 574 | 12.1 | 0.19 | 0.589 |
|  | Villamaría | Cauca-Valley Montane | 438 | -9.0 | -0.13 | 0.781 |
|  | Viterbo | Magdalena Valley Dry | 122 | 4.1 | 0.40 | 0.256 |
| **Caquetá** | Albania | Caquetá Moist | 400 | 0.0 | 0.07 | 0.856 |
|  | Belén de los Andaquíes | Cordillera Oriental | 989 | 42.2 | 0.30 | 0.403 |
|  | Cartagena del Chairá | Caquetá Moist | 13,222 | -239.1 | -0.16 | 0.649 |
|  | Curillo | Caquetá Moist | 473 | -13.6 | -0.27 | 0.448 |
|  | El Doncello | Caquetá Moist | 1,087 | 6.9 | 0.13 | 0.731 |
|  | El Paujil | Caquetá Moist | 1,396 | -0.9 | -0.01 | 0.981 |
|  | Florencia | Cordillera Oriental | 2,306 | 211.4 | 0.43 | 0.330 |
|  | La Montañita | Caquetá Moist | 1,945 | -21.8 | -0.19 | 0.605 |
|  | Milán | Caquetá Moist | 1,354 | -11.9 | -0.14 | 0.694 |
|  | Morelia | Caquetá Moist | 460 | -3.0 | -0.13 | 0.712 |
|  | Puerto Rico | Cordillera Oriental | 3,097 | 90.5 | 0.36 | 0.380 |
|  | San José del Fragua | Northern Andean | 1,176 | 99.7 | 0.42 | 0.350 |
|  | San Vicente del Caguán | Caquetá Moist | 18,527 | -687.9 | -0.46 | 0.179 |
|  | Solano | Caquetá Moist | 42,615 | -116.3 | -0.09 | 0.799 |
|  | Solita | Caquetá Moist | 878 | -6.2 | -0.09 | 0.814 |
|  | Valparaiso | Caquetá Moist | 998 | -10.8 | -0.45 | 0.190 |
| **Casanare** | Aguazul | Llanos | 1,456 | -90.5 | -0.43 | 0.219 |
|  | Chámeza | Cordillera Oriental | 321 | -33.4 | -0.30 | 0.624 |
|  | Hato Corozal | Llanos | 5,845 | -116.8 | -0.51 | 0.136 |
|  | La Salina | Cordillera Oriental | 209 | -10.8 | -0.38 | 0.274 |
|  | Mani | Llanos | 3,760 | -46.3 | -0.43 | 0.217 |
|  | Monterrey | Apure-Villavicencio | 759 | -1.2 | -0.03 | 0.930 |
|  | Nunchía | Apure-Villavicencio | 1,139 | -9.7 | -0.30 | 0.408 |
|  | Orocue | Llanos | 4,762 | -8.0 | -0.11 | 0.756 |
|  | Paz de Ariporo | Llanos | 12,084 | -127.6 | -0.56 | 0.094 |
|  | Pore | Llanos | 782 | -58.3 | -0.73 | 0.018 |
|  | Recetor | Cordillera Oriental | 184 | -0.8 | -0.03 | 0.940 |
|  | Sabanalarga | Apure-Villavicencio | 406 | 10.4 | 0.32 | 0.360 |
|  | Sacama | Cordillera Oriental | 321 | -35.8 | -0.75 | 0.012 |
|  | San Luis de Palenque | Llanos | 3,006 | -104.1 | -0.62 | 0.058 |
|  | Támara | Cordillera Oriental | 1,045 | -46.9 | -0.35 | 0.315 |
|  | Tauramena | Llanos | 2,410 | -22.4 | -0.12 | 0.734 |
|  | Trinidad | Llanos | 2,973 | -120.6 | -0.74 | 0.014 |
|  | Villanueva | Llanos | 800 | -17.2 | -0.21 | 0.566 |
|  | Yopal | Llanos | 2,518 | -144.5 | -0.43 | 0.216 |
| **Cauca** | Almaguer | Northwestern Andean | 244 | 30.4 | 0.60 | 0.070 |
|  | Argelia | Northwestern Andean | 706 | 43.8 | 0.38 | 0.280 |
|  | Balboa | Northwestern Andean | 414 | 41.3 | 0.65 | 0.042 |
|  | Bolívar | Northwestern Andean | 815 | 24.1 | 0.62 | 0.058 |
|  | Buenos Aires | Cauca-Valley Montane | 354 | 7.8 | 0.19 | 0.592 |
|  | Cajibío | Cauca-Valley Montane | 560 | 60.2 | 0.60 | 0.066 |
|  | Caldono | Cauca-Valley Montane | 351 | 7.7 | 0.15 | 0.672 |
|  | Caloto | Cauca-Valley Montane | 437 | 1.9 | 0.08 | 0.836 |
|  | Corinto | Cauca-Valley Montane | 320 | 34.8 | 0.61 | 0.147 |
|  | El Tambo | Northwestern Andean | 2,818 | 165.2 | 0.29 | 0.421 |
|  | Florencia | Northwestern Andean | 62 | 6.4 | 0.60 | 0.068 |
|  | Guapi | Chocó-Darién Moist | 2,939 | -130.0 | -0.18 | 0.729 |
|  | Inzá | Northern Andean | 728 | 37.8 | 0.33 | 0.465 |
|  | Jambaló | Cauca-Valley Montane | 255 | 13.4 | 0.37 | 0.287 |
|  | La Sierra | Northwestern Andean | 211 | 21.6 | 0.50 | 0.137 |
|  | La Vega | Northwestern Andean | 541 | 72.6 | 0.64 | 0.044 |
|  | López | Chocó-Darién Moist | 3,225 | -9.1 | -0.02 | 0.965 |
|  | Mercaderes | Magdalena Valley Dry | 741 | 2.3 | 0.38 | 0.274 |
|  | Miranda | Cauca-Valley Montane | 195 | 10.3 | 0.65 | 0.113 |
|  | Morales | Cauca-Valley Montane | 458 | 39.3 | 0.33 | 0.347 |
|  | Padilla | Magdalena Valley Dry | 71 | 2.5 | 0.42 | 0.231 |
|  | Paez | Northern Andean | 1,663 | 97.9 | 0.73 | 0.158 |
|  | Patía | Magdalena Valley Dry | 776 | 24.4 | 0.68 | 0.032 |
|  | Piamonte | Northern Andean | 1,149 | 12.0 | 0.05 | 0.901 |
|  | Piendamó | Cauca-Valley Montane | 182 | 8.8 | 0.20 | 0.576 |
|  | Popayán | Cauca-Valley Montane | 491 | 28.3 | 0.60 | 0.066 |
|  | Puerto Tejada | Magdalena Valley Dry | 109 | 1.4 | 0.19 | 0.604 |
|  | Puracé | Cauca-Valley Montane | 520 | -2.8 | -0.05 | 0.881 |
|  | Rosas | Northwestern Andean | 146 | 17.5 | 0.51 | 0.133 |
|  | San Sebastián | Northern Andean | 405 | 20.7 | 0.61 | 0.060 |
|  | Santa Rosa | Northern Andean | 3,051 | 324.2 | 0.78 | 0.118 |
|  | Santander de Quilichao | Cauca-Valley Montane | 461 | 5.1 | 0.33 | 0.351 |
|  | Silvia | Cauca-Valley Montane | 683 | 5.2 | 0.15 | 0.673 |
|  | Sotara | Northwestern Andean | 464 | 64.0 | 0.71 | 0.023 |
|  | Suárez | Cauca-Valley Montane | 447 | -5.2 | -0.07 | 0.847 |
|  | Sucre | Northwestern Andean | 136 | 11.7 | 0.45 | 0.197 |
|  | Timbío | Northwestern Andean | 171 | 14.2 | 0.62 | 0.058 |
|  | Timbiquí | Chocó-Darién Moist | 2,051 | 58.7 | 0.14 | 0.737 |
|  | Toribio | Cauca-Valley Montane | 450 | -30.1 | -0.37 | 0.292 |
|  | Totoró | Cauca-Valley Montane | 438 | 18.4 | 0.73 | 0.018 |
|  | Villa Rica | Magdalena Valley Dry | 95 | -0.1 | -0.04 | 0.911 |
| **Cesar** | Aguachica | Mag-Urabá Moist | 875 | 28.6 | 0.63 | 0.051 |
|  | Agustín Codazzi | Cordillera Oriental | 1,775 | 130.1 | 0.67 | 0.033 |
|  | Astrea | Sinú-Valley Dry | 612 | 4.0 | 0.05 | 0.894 |
|  | Becerril | Sinú-Valley Dry | 1,251 | 165.9 | 0.59 | 0.072 |
|  | Bosconia | Sinú-Valley Dry | 590 | 24.3 | 0.31 | 0.388 |
|  | Chimichagua | Mag-Urabá Moist | 1,392 | 38.3 | 0.30 | 0.395 |
|  | Chiriguaná | Sinú-Valley Dry | 1,176 | 65.0 | 0.26 | 0.475 |
|  | Curumaní | Sinú-Valley Dry | 938 | 35.7 | 0.36 | 0.314 |
|  | El Copey | Sinú-Valley Dry | 970 | -13.3 | -0.07 | 0.853 |
|  | El Paso | Sinú-Valley Dry | 808 | 33.3 | 0.27 | 0.443 |
|  | Gamarra | Mag-Urabá Moist | 332 | 3.8 | 0.62 | 0.057 |
|  | González | Cordillera Oriental | 53 | 2.3 | 0.19 | 0.602 |
|  | La Gloria | Mag-Urabá Moist | 833 | 103.4 | 0.75 | 0.012 |
|  | La Jagua de Ibirico | Sinú-Valley Dry | 764 | 81.1 | 0.59 | 0.070 |
|  | La Paz | Cordillera Oriental | 1,159 | 74.8 | 0.59 | 0.073 |
|  | Manaure | Cordillera Oriental | 167 | 6.9 | 0.31 | 0.388 |
|  | Pailitas | Northern Andean | 485 | 38.3 | 0.57 | 0.085 |
|  | Pelaya | Northern Andean | 352 | 43.6 | 0.70 | 0.025 |
|  | Pueblo Bello | Cordillera Oriental | 737 | -45.9 | -0.38 | 0.281 |
|  | Río de Oro | Mag-Urabá Moist | 443 | 10.6 | 0.64 | 0.044 |
|  | San Alberto | Mag-Urabá Moist | 569 | -3.5 | -0.07 | 0.839 |
|  | San Diego | Guajira Xeric | 654 | 22.8 | 0.58 | 0.081 |
|  | San Martín | Mag-Urabá Moist | 992 | -11.2 | -0.25 | 0.491 |
|  | Tamalameque | Mag-Urabá Moist | 611 | 25.9 | 0.53 | 0.114 |
|  | Valledupar | Sinú-Valley Dry | 4,195 | -6.0 | -0.01 | 0.976 |
| **Chocó** | Acandí | Chocó-Darién Moist | 892 | -118.7 | -0.58 | 0.098 |
|  | Alto Baudó | Chocó-Darién Moist | 1,809 | -125.7 | -0.18 | 0.636 |
|  | Atrato | Chocó-Darién Moist | 456 | 110.1 | 0.51 | 0.133 |
|  | Bagadó | Northwestern Andean | 778 | 104.0 | 0.36 | 0.432 |
|  | Bahía Solano | Chocó-Darién Moist | 1,075 | -51.3 | -0.43 | 0.475 |
|  | Bajo Baudó | Chocó-Darién Moist | 3,387 | 233.0 | 0.43 | 0.294 |
|  | Belén de Bajirá | Chocó-Darién Moist | 1,123 | -43.3 | -0.24 | 0.502 |
|  | Bojayá | Chocó-Darién Moist | 3,492 | -205.6 | -0.19 | 0.634 |
|  | Carmen del Darién | Chocó-Darién Moist | 3,311 | 63.8 | 0.06 | 0.861 |
|  | Cértegui | Chocó-Darién Moist | 238 | 22.7 | 0.24 | 0.537 |
|  | Condoto | Northwestern Andean | 824 | -15.4 | -0.09 | 0.886 |
|  | El Cantón de San Pablo | Chocó-Darién Moist | 420 | 105.0 | 0.60 | 0.067 |
|  | El Carmen de Atrato | Northwestern Andean | 966 | -149.4 | -0.50 | 0.387 |
|  | Istmina | Chocó-Darién Moist | 1,970 | 144.3 | 0.40 | 0.248 |
|  | Juradó | Chocó-Darién Moist | 1,327 | -23.8 | -0.22 | 0.539 |
|  | Litoral de San Juan | Chocó-Darién Moist | 3,806 | 227.1 | 0.33 | 0.391 |
|  | Lloró | Chocó-Darién Moist | 802 | 322.4 | 0.74 | 0.056 |
|  | Medio Atrato | Chocó-Darién Moist | 1,756 | 154.9 | 0.31 | 0.419 |
|  | Medio Baudó | Chocó-Darién Moist | 1,428 | 343.8 | 0.64 | 0.047 |
|  | Medio San Juan | Chocó-Darién Moist | 706 | 18.3 | 0.11 | 0.758 |
|  | Novita | Chocó-Darién Moist | 1,168 | -114.6 | -0.29 | 0.810 |
|  | Nuquí | Chocó-Darién Moist | 1,007 | -22.9 | -0.14 | 0.734 |
|  | Quibdó | Chocó-Darién Moist | 3,234 | -9.0 | -0.01 | 0.984 |
|  | Río Iró | Chocó-Darién Moist | 123 | 7.4 | 0.15 | 0.708 |
|  | Río Quito | Chocó-Darién Moist | 708 | 91.9 | 0.29 | 0.420 |
|  | Riosucio | Chocó-Darién Moist | 5,892 | -38.9 | -0.05 | 0.900 |
|  | San José del Palmar | Northwestern Andean | 1,051 | 250.9 | 0.65 | 0.060 |
|  | Sipí | Northwestern Andean | 1,306 | 165.6 | 0.41 | 0.493 |
|  | Tadó | Northwestern Andean | 846 | 529.1 | 0.87 | 0.130 |
|  | Unguía | Chocó-Darién Moist | 1,318 | -21.7 | -0.16 | 0.656 |
|  | Unión Panamerica | Chocó-Darién Moist | 192 | 21.6 | 0.26 | 0.461 |
| **Córdoba** | Ayapel | Mag-Urabá Moist | 1,939 | 69.8 | 0.72 | 0.020 |
|  | Buenavista | Mag-Urabá Moist | 832 | -0.5 | -0.08 | 0.816 |
|  | Canalete | Mag-Urabá Moist | 340 | 0.3 | 0.05 | 0.901 |
|  | Cereté | Mag-Urabá Moist | 271 | 0.0 | 0.03 | 0.934 |
|  | Chimá | Mag-Urabá Moist | 329 | -8.5 | -0.44 | 0.209 |
|  | Chinú | Mag-Urabá Moist | 597 | 0.5 | 0.53 | 0.115 |
|  | Ciénaga de Oro | Mag-Urabá Moist | 650 | 2.4 | 0.64 | 0.045 |
|  | Cotorra | Mag-Urabá Moist | 81 | 1.0 | 0.48 | 0.158 |
|  | La Apartada | Mag-Urabá Moist | 255 | 1.2 | 0.59 | 0.075 |
|  | Lorica | Mag-Urabá Moist | 900 | -5.1 | -0.28 | 0.440 |
|  | Los Córdobas | Mag-Urabá Moist | 468 | 4.6 | 0.71 | 0.021 |
|  | Momil | Mag-Urabá Moist | 158 | -5.6 | -0.84 | 0.002 |
|  | Moñitos | Mag-Urabá Moist | 213 | 2.4 | 0.48 | 0.338 |
|  | Montelíbano | Mag-Urabá Moist | 1,801 | 47.5 | 0.34 | 0.331 |
|  | Montería | Mag-Urabá Moist | 3,014 | 1.3 | 0.06 | 0.869 |
|  | Planeta Rica | Mag-Urabá Moist | 1,227 | 0.1 | 0.01 | 0.980 |
|  | Pueblo Nuevo | Mag-Urabá Moist | 817 | 8.9 | 0.73 | 0.016 |
|  | Puerto Escondido | Mag-Urabá Moist | 436 | 2.1 | 0.42 | 0.261 |
|  | Puerto Libertador | Northwestern Andean | 2,051 | -12.3 | -0.05 | 0.881 |
|  | Purísima | Mag-Urabá Moist | 123 | -0.2 | -0.17 | 0.638 |
|  | Sahagún | Mag-Urabá Moist | 976 | -0.1 | -0.59 | 0.075 |
|  | San Andrés Sotavento | Mag-Urabá Moist | 319 | 0.0 | 0.17 | 0.631 |
|  | San Antero | Mag-Urabá Moist | 211 | 6.1 | 0.60 | 0.066 |
|  | San Bernardo del Viento | Mag-Urabá Moist | 330 | 4.2 | 0.39 | 0.293 |
|  | San Carlos | Mag-Urabá Moist | 506 | 0.3 | 0.31 | 0.383 |
|  | San Pelayo | Mag-Urabá Moist | 487 | -0.1 | -0.20 | 0.580 |
|  | Tierralta | Northwestern Andean | 4,885 | 27.8 | 0.06 | 0.864 |
|  | Valencia | Mag-Urabá Moist | 994 | 27.3 | 0.27 | 0.458 |
| **Cundina-marca** | Agua de Dios | Magdalena Valley Dry | 73 | 2.2 | 0.11 | 0.763 |
|  | Albán | Northern Andean | 58 | 5.3 | 0.25 | 0.485 |
|  | Anapoima | Magdalena Valley Dry | 127 | 6.0 | 0.12 | 0.747 |
|  | Anolaima | Northern Andean | 116 | 1.0 | 0.09 | 0.883 |
|  | Apulo | Magdalena Valley Dry | 121 | 11.5 | 0.23 | 0.524 |
|  | Arbeláez | Northern Andean | 162 | 18.1 | 0.90 | 0.040 |
|  | Beltrán | Magdalena Valley Dry | 189 | -7.1 | -0.30 | 0.399 |
|  | Bituima | Northern Andean | 57 | 6.3 | 0.63 | 0.053 |
|  | Bojacá | Northern Andean | 103 | 1.3 | 0.35 | 0.493 |
|  | Cachipay | Northern Andean | 61 | 5.1 | 0.38 | 0.320 |
|  | Cajicá | Northern Andean | 56 | 3.4 | 0.74 | 0.015 |
|  | Caparrapí | Northern Andean | 619 | 61.7 | 0.68 | 0.030 |
|  | Caqueza | Cordillera Oriental | 109 | 0.1 | 0.17 | 0.631 |
|  | Carmen de Carupa | Northern Andean | 319 | 4.7 | 0.36 | 0.303 |
|  | Chaguaní | Magdalena Valley Dry | 173 | 4.1 | 0.09 | 0.806 |
|  | Chía | Northern Andean | 80 | 1.9 | 0.47 | 0.175 |
|  | Chipaque | Cordillera Oriental | 117 | 0.1 | 0.01 | 0.976 |
|  | Choachí | Cordillera Oriental | 215 | 0.7 | 0.05 | 0.893 |
|  | Chocontá | Northern Andean | 309 | 0.0 | 0.00 | 0.995 |
|  | Cogua | Northern Andean | 132 | -0.7 | -0.20 | 0.574 |
|  | Cota | Northern Andean | 57 | 4.2 | 0.58 | 0.078 |
|  | Cucunubá | Northern Andean | 113 | 0.9 | 0.73 | 0.018 |
|  | El Colegio | Northern Andean | 119 | 16.3 | 0.67 | 0.142 |
|  | El Peñón | Northern Andean | 135 | 39.2 | 0.80 | 0.006 |
|  | El Rosal | Northern Andean | 84 | -0.7 | -0.24 | 0.509 |
|  | Facatativá | Northern Andean | 157 | 1.9 | 0.17 | 0.636 |
|  | Fómeque | Cordillera Oriental | 472 | 4.9 | 0.18 | 0.616 |
|  | Fosca | Cordillera Oriental | 114 | 0.0 | 0.00 | 0.998 |
|  | Fúquene | Northern Andean | 82 | 7.5 | 0.86 | 0.002 |
|  | Fusagasugá | Northern Andean | 199 | 10.6 | 0.81 | 0.014 |
|  | Gachalá | Cordillera Oriental | 385 | 22.8 | 0.50 | 0.141 |
|  | Gachancipá | Northern Andean | 47 | 1.0 | 0.41 | 0.240 |
|  | Gachetá | Cordillera Oriental | 263 | 3.7 | 0.18 | 0.613 |
|  | Gama | Cordillera Oriental | 110 | -2.1 | -0.16 | 0.665 |
|  | Girardot | Magdalena Valley Dry | 133 | -5.9 | -0.18 | 0.613 |
|  | Granada | Northern Andean | 50 | 0.7 | 0.81 | 0.096 |
|  | Guachetá | Northern Andean | 198 | 7.2 | 0.67 | 0.032 |
|  | Guaduas | Magdalena Valley Dry | 772 | -16.9 | -0.10 | 0.781 |
|  | Guasca | Northern Andean | 345 | 8.4 | 0.56 | 0.090 |
|  | Guataquí | Magdalena Valley Dry | 88 | -16.2 | -0.59 | 0.075 |
|  | Guatavita | Northern Andean | 246 | 6.6 | 0.81 | 0.005 |
|  | Guayabal de Siquima | Northern Andean | 65 | 22.8 | 0.70 | 0.023 |
|  | Guayabetal | Cordillera Oriental | 219 | 3.3 | 0.08 | 0.868 |
|  | Gutiérrez | Cordillera Oriental | 473 | -2.5 | -0.08 | 0.824 |
|  | Jerusalén | Magdalena Valley Dry | 229 | -21.9 | -0.29 | 0.414 |
|  | Junín | Cordillera Oriental | 343 | 12.2 | 0.37 | 0.290 |
|  | La Calera | Northern Andean | 339 | 1.7 | 0.07 | 0.848 |
|  | La Mesa | Northern Andean | 157 | 16.4 | 0.74 | 0.015 |
|  | La Palma | Northern Andean | 187 | 55.3 | 0.74 | 0.015 |
|  | La Peña | Magdalena Valley Dry | 128 | 15.5 | 0.68 | 0.032 |
|  | La Vega | Northern Andean | 160 | 20.6 | 0.68 | 0.030 |
|  | Lenguazaque | Northern Andean | 152 | 3.2 | 0.68 | 0.029 |
|  | Machetá | Cordillera Oriental | 223 | -3.3 | -0.20 | 0.581 |
|  | Madrid | Northern Andean | 126 | 0.9 | 0.45 | 0.196 |
|  | Manta | Cordillera Oriental | 114 | 0.6 | 0.07 | 0.856 |
|  | Medina | Cordillera Oriental | 1,197 | 80.8 | 0.59 | 0.165 |
|  | Mosquera | Northern Andean | 105 | 0.8 | 0.54 | 0.104 |
|  | Nariño | Magdalena Valley Dry | 56 | -7.0 | -0.45 | 0.193 |
|  | Nemocón | Northern Andean | 100 | 2.1 | 0.52 | 0.125 |
|  | Nilo | Magdalena Valley Dry | 232 | 0.5 | 0.01 | 0.987 |
|  | Nimaima | Magdalena Valley Dry | 65 | -2.0 | -0.23 | 0.520 |
|  | Nocaima | Northern Andean | 68 | 12.9 | 0.54 | 0.107 |
|  | Pacho | Northern Andean | 413 | 40.2 | 0.79 | 0.011 |
|  | Paime | Northern Andean | 179 | 64.4 | 0.83 | 0.003 |
|  | Pandi | Northern Andean | 88 | 18.9 | 0.59 | 0.073 |
|  | Paratebueno | Apure-Villavicencio | 922 | 11.7 | 0.61 | 0.059 |
|  | Pasca | Northern Andean | 258 | 9.1 | 0.76 | 0.446 |
|  | Puerto Salgar | Mag-Urabá Moist | 515 | 29.0 | 0.91 | 0.000 |
|  | Puli | Magdalena Valley Dry | 194 | -5.2 | -0.09 | 0.794 |
|  | Quebrada Negra | Magdalena Valley Dry | 79 | 8.0 | 0.28 | 0.426 |
|  | Quetame | Cordillera Oriental | 146 | -1.6 | -0.06 | 0.868 |
|  | Quipile | Northern Andean | 127 | 5.4 | 0.23 | 0.514 |
|  | Ricaurte | Magdalena Valley Dry | 135 | -0.1 | 0.00 | 0.993 |
|  | San Antonio del Tequendama | Northern Andean | 92 | 15.7 | 0.95 | 0.004 |
|  | San Bernardo | Northern Andean | 232 | 4.3 | 0.53 | 0.641 |
|  | San Cayetano | Northern Andean | 314 | 49.8 | 0.72 | 0.018 |
|  | San Francisco | Northern Andean | 117 | -1.0 | -0.10 | 0.807 |
|  | San Juan de Ríoseco | Magdalena Valley Dry | 322 | 6.2 | 0.08 | 0.832 |
|  | Sasaima | Northern Andean | 104 | 12.0 | 0.31 | 0.423 |
|  | Sesquilé | Northern Andean | 138 | 1.5 | 0.34 | 0.343 |
|  | Sibaté | Northern Andean | 118 | 0.3 | 0.14 | 0.796 |
|  | Silvania | Northern Andean | 173 | 5.6 | 0.72 | 0.280 |
|  | Simijaca | Northern Andean | 110 | 0.7 | 0.53 | 0.111 |
|  | Soacha | Northern Andean | 187 | 1.2 | 0.33 | 0.349 |
|  | Sopo | Northern Andean | 116 | 3.7 | 0.64 | 0.048 |
|  | Subachoque | Northern Andean | 214 | 0.8 | 0.16 | 0.659 |
|  | Suesca | Northern Andean | 182 | 0.0 | 0.35 | 0.324 |
|  | Supatá | Northern Andean | 133 | 16.3 | 0.77 | 0.015 |
|  | Susa | Northern Andean | 106 | 1.5 | 0.65 | 0.042 |
|  | Sutatausa | Northern Andean | 60 | 0.6 | 0.41 | 0.234 |
|  | Tabio | Northern Andean | 71 | 3.4 | 0.82 | 0.003 |
|  | Tausa | Northern Andean | 202 | -0.2 | -0.05 | 0.898 |
|  | Tena | Northern Andean | 51 | 1.7 | 0.63 | 0.091 |
|  | Tenjo | Northern Andean | 113 | 2.7 | 0.51 | 0.133 |
|  | Tibacuy | Northern Andean | 87 | 15.9 | 0.81 | 0.008 |
|  | Tibirita | Cordillera Oriental | 59 | 0.0 | -0.01 | 0.984 |
|  | Tocaima | Magdalena Valley Dry | 253 | 0.2 | 0.00 | 0.994 |
|  | Tocancipá | Northern Andean | 68 | 1.3 | 0.80 | 0.005 |
|  | Topaipí | Northern Andean | 148 | 43.6 | 0.76 | 0.011 |
|  | Ubalá | Cordillera Oriental | 529 | 19.9 | 0.33 | 0.351 |
|  | Ubaque | Cordillera Oriental | 121 | -2.7 | -0.42 | 0.229 |
|  | Une | Cordillera Oriental | 230 | -1.4 | -0.27 | 0.456 |
|  | Útica | Magdalena Valley Dry | 92 | 0.8 | 0.04 | 0.922 |
|  | Venecia | Northern Andean | 109 | 22.5 | 0.85 | 0.014 |
|  | Vergara | Northern Andean | 146 | 31.5 | 0.70 | 0.025 |
|  | Viani | Northern Andean | 70 | 6.4 | 0.36 | 0.308 |
|  | Villa de San Diego de Ubaté | Northern Andean | 108 | 0.4 | 0.76 | 0.011 |
|  | Villagómez | Northern Andean | 63 | 8.6 | 0.37 | 0.291 |
|  | Villapinzón | Northern Andean | 216 | -0.4 | -0.40 | 0.254 |
|  | Villeta | Northern Andean | 139 | 7.3 | 0.46 | 0.186 |
|  | Viotá | Northern Andean | 204 | 32.5 | 0.54 | 0.209 |
|  | Yacopí | Northern Andean | 954 | 108.9 | 0.70 | 0.023 |
|  | Zipacón | Northern Andean | 55 | 1.6 | 0.58 | 0.224 |
|  | Zipaquirá | Northern Andean | 185 | 1.6 | 0.29 | 0.412 |
| **Guainía** | Barranco Mina | Caquetá Moist | 9,553 | 39.4 | 0.21 | 0.559 |
|  | Cacahual | Caquetá Moist | 2,313 | -27.6 | -0.09 | 0.799 |
|  | Guadalupe | Caquetá Moist | 1,168 | 2.5 | 0.07 | 0.855 |
|  | Inírida | Caquetá Moist | 15,845 | 91.8 | 0.15 | 0.684 |
|  | Mapiripana | Caquetá Moist | 4,734 | 33.3 | 0.24 | 0.500 |
|  | Morichal Nuevo | Caquetá Moist | 8,588 | -13.5 | -0.07 | 0.848 |
|  | Pana Pana (Campo Alegre) | Caquetá Moist | 10,117 | 8.3 | 0.06 | 0.878 |
|  | Puerto Colombia | Caquetá Moist | 15,741 | -56.1 | -0.14 | 0.705 |
|  | San Felipe | Caquetá Moist | 3,054 | 22.0 | 0.16 | 0.658 |
| **Guaviare** | Calamar | Caquetá Moist | 14,054 | 245.5 | 0.18 | 0.621 |
|  | El Retorno | Caquetá Moist | 12,328 | 83.0 | 0.08 | 0.833 |
|  | Miraflores | Caquetá Moist | 12,749 | 1021.0 | 0.56 | 0.092 |
|  | San José del Guaviare | Caquetá Moist | 16,346 | 261.1 | 0.23 | 0.517 |
| **Huila** | Acevedo | Northern Andean | 672 | 45.7 | 0.61 | 0.107 |
|  | Agrado | Magdalena Valley Dry | 268 | 0.9 | 0.43 | 0.212 |
|  | Aípe | Magdalena Valley Dry | 820 | 51.5 | 0.52 | 0.127 |
|  | Algeciras | Northern Andean | 628 | 40.4 | 0.31 | 0.452 |
|  | Altamira | Magdalena Valley Dry | 199 | 0.9 | 0.58 | 0.076 |
|  | Baraya | Northern Andean | 684 | 58.0 | 0.63 | 0.052 |
|  | Campoalegre | Magdalena Valley Dry | 473 | 19.4 | 0.50 | 0.145 |
|  | Colombia | Northern Andean | 1,853 | 47.0 | 0.14 | 0.792 |
|  | Elias | Northern Andean | 77 | 3.2 | 0.49 | 0.152 |
|  | Garzón | Northern Andean | 624 | 113.9 | 0.95 | 0.003 |
|  | Gigante | Magdalena Valley Dry | 518 | 25.0 | 0.40 | 0.322 |
|  | Guadalupe | Northern Andean | 227 | 33.5 | 0.64 | 0.048 |
|  | Hobo | Magdalena Valley Dry | 208 | 9.1 | 0.57 | 0.088 |
|  | Íquira | Northern Andean | 532 | 29.7 | 0.38 | 0.401 |
|  | Isnos | Northern Andean | 658 | 66.8 | 0.61 | 0.109 |
|  | La Argentina | Northern Andean | 395 | 41.9 | 0.40 | 0.371 |
|  | La Plata | Northern Andean | 1,235 | 140.4 | 0.64 | 0.172 |
|  | Nataga | Northern Andean | 173 | 44.6 | 0.75 | 0.012 |
|  | Neiva | Northern Andean | 1,348 | 87.6 | 0.65 | 0.060 |
|  | Oporapa | Northern Andean | 143 | 2.6 | 0.08 | 0.834 |
|  | Paicol | Magdalena Valley Dry | 312 | 0.9 | 0.52 | 0.123 |
|  | Palermo | Magdalena Valley Dry | 832 | 8.5 | 0.63 | 0.053 |
|  | Palestina | Northern Andean | 244 | 18.3 | 0.22 | 0.573 |
|  | Pital | Northern Andean | 196 | 9.8 | 0.44 | 0.201 |
|  | Pitalito | Northern Andean | 579 | 44.3 | 0.44 | 0.241 |
|  | Rivera | Magdalena Valley Dry | 354 | 40.1 | 0.67 | 0.071 |
|  | Salado Blanco | Northern Andean | 297 | 17.0 | 0.39 | 0.339 |
|  | San Agustin | Northern Andean | 1,556 | 5.8 | 0.07 | 0.932 |
|  | Santa María | Northern Andean | 359 | 38.5 | 0.56 | 0.116 |
|  | Suazá | Northern Andean | 339 | 31.2 | 0.66 | 0.039 |
|  | Tarquí | Northern Andean | 334 | 18.8 | 0.59 | 0.073 |
|  | Tello | Magdalena Valley Dry | 567 | 68.4 | 0.47 | 0.168 |
|  | Teruel | Northern Andean | 534 | 8.9 | 0.07 | 0.877 |
|  | Tesalia | Northern Andean | 336 | 0.5 | 0.33 | 0.354 |
|  | Timaná | Northern Andean | 190 | 25.3 | 0.50 | 0.140 |
|  | Villa Vieja | Magdalena Valley Dry | 534 | 6.0 | 0.19 | 0.685 |
|  | Yaguara | Magdalena Valley Dry | 323 | 0.1 | 0.48 | 0.160 |
| **La Guajira** | Albania | Guajira Xeric | 594 | 70.4 | 0.35 | 0.327 |
|  | Barrancas | Cordillera Oriental | 947 | 41.8 | 0.26 | 0.468 |
|  | Dibulla | Cordillera Oriental | 1,811 | -45.7 | -0.27 | 0.451 |
|  | Distracción | Guajira Xeric | 220 | 12.0 | 0.22 | 0.548 |
|  | El Molino | Cordillera Oriental | 232 | 10.3 | 0.39 | 0.270 |
|  | Fonseca | Cordillera Oriental | 662 | 32.3 | 0.24 | 0.504 |
|  | Hato Nuevo | Guajira Xeric | 217 | 57.6 | 0.71 | 0.023 |
|  | La Jagua del Pilar | Cordillera Oriental | 224 | 15.2 | 0.66 | 0.038 |
|  | Maicao | Guajira Xeric | 1,747 | 240.5 | 0.56 | 0.093 |
|  | Manaure | Guajira Xeric | 1,628 | 89.1 | 0.59 | 0.075 |
|  | Riohacha | Guajira Xeric | 3,027 | 366.1 | 0.43 | 0.210 |
|  | San Juan del Cesar | Guajira Xeric | 1,453 | 24.0 | 0.09 | 0.804 |
|  | Uribia | Guajira Xeric | 7,890 | 589.2 | 0.68 | 0.031 |
|  | Urumita | Cordillera Oriental | 303 | 23.8 | 0.58 | 0.080 |
|  | Villanueva | Cordillera Oriental | 286 | 11.8 | 0.53 | 0.118 |
| **Magdalena** | Algarrobo | Sinú-Valley Dry | 416 | 68.9 | 0.57 | 0.084 |
|  | Aracataca | Sinú-Valley Dry | 1,708 | -24.9 | -0.19 | 0.595 |
|  | Ariguaní | Sinú-Valley Dry | 1,164 | 102.0 | 0.54 | 0.107 |
|  | Cerro de San Antonio | Sinú-Valley Dry | 177 | 25.5 | 0.54 | 0.106 |
|  | Chivolo | Sinú-Valley Dry | 533 | -19.3 | -0.21 | 0.568 |
|  | Ciénaga | Cordillera Oriental | 1,327 | -23.4 | -0.24 | 0.510 |
|  | Concordia | Sinú-Valley Dry | 117 | 9.3 | 0.52 | 0.124 |
|  | El Banco | Mag-Urabá Moist | 818 | -12.5 | -0.27 | 0.455 |
|  | El Piñón | Sinú-Valley Dry | 555 | 98.6 | 0.63 | 0.053 |
|  | El Retén | Sinú-Valley Dry | 275 | 15.5 | 0.26 | 0.475 |
|  | Fundación | Sinú-Valley Dry | 1,002 | -32.7 | -0.19 | 0.604 |
|  | Guamal | Sinú-Valley Dry | 535 | -9.1 | -0.07 | 0.858 |
|  | Nueva Granada | Sinú-Valley Dry | 858 | 24.0 | 0.27 | 0.452 |
|  | Pedraza | Sinú-Valley Dry | 313 | 25.2 | 0.49 | 0.154 |
|  | Pijiño del Carmen | Sinú-Valley Dry | 686 | 22.5 | 0.16 | 0.667 |
|  | Pivijay | Sinú-Valley Dry | 1,648 | 179.4 | 0.38 | 0.274 |
|  | Plato | Mag-Urabá Moist | 1,443 | -7.0 | -0.17 | 0.648 |
|  | Puebloviejo | Guajira Xeric | 660 | 40.9 | 0.68 | 0.137 |
|  | Remolino | Sinú-Valley Dry | 592 | 48.7 | 0.44 | 0.208 |
|  | Salamina | Sinú-Valley Dry | 172 | 33.0 | 0.64 | 0.044 |
|  | San Ángel | Sinú-Valley Dry | 1,242 | 100.5 | 0.28 | 0.437 |
|  | San Sebastián de Buenavista | Sinú-Valley Dry | 448 | 4.1 | 0.04 | 0.914 |
|  | San Zenón | Mag-Urabá Moist | 248 | 8.4 | 0.46 | 0.183 |
|  | Santa Ana | Sinú-Valley Dry | 1,121 | 65.9 | 0.24 | 0.499 |
|  | Santa Bárbara de Pinto | Mag-Urabá Moist | 487 | 3.1 | 0.24 | 0.507 |
|  | Santa Marta | Sinú-Valley Dry | 2,344 | 16.9 | 0.10 | 0.777 |
|  | Sitionuevo | Guajira Xeric | 991 | 106.0 | 0.58 | 0.079 |
|  | Tenerife | Mag-Urabá Moist | 498 | -1.3 | -0.20 | 0.586 |
|  | Zapayán | Sinú-Valley Dry | 356 | 23.2 | 0.42 | 0.221 |
|  | Zona Bananera | Sinú-Valley Dry | 446 | 30.9 | 0.29 | 0.408 |
| **Meta** | Acacias | Cordillera Oriental | 1,247 | 36.8 | 0.43 | 0.286 |
|  | Barranca de Upía | Llanos | 436 | 14.7 | 0.29 | 0.414 |
|  | Cabuyaro | Llanos | 919 | 33.1 | 0.52 | 0.121 |
|  | Castilla la Nueva | Llanos | 501 | 70.8 | 0.82 | 0.004 |
|  | Cubarral | Cordillera Oriental | 1,234 | 38.3 | 0.34 | 0.461 |
|  | Cumaral | Apure-Villavicencio | 634 | -6.4 | -0.41 | 0.245 |
|  | El Calvario | Cordillera Oriental | 281 | 49.3 | 0.47 | 0.292 |
|  | El Castillo | Cordillera Oriental | 654 | 20.5 | 0.30 | 0.512 |
|  | El Dorado | Apure-Villavicencio | 145 | 9.3 | 0.69 | 0.028 |
|  | Fuente de Oro | Apure-Villavicencio | 527 | -1.0 | -0.11 | 0.767 |
|  | Granada | Apure-Villavicencio | 393 | 0.1 | 0.29 | 0.420 |
|  | Guamal | Cordillera Oriental | 622 | 28.7 | 0.63 | 0.093 |
|  | La Macarena | Caquetá Moist | 10,756 | -711.6 | -0.62 | 0.055 |
|  | Lejanías | Cordillera Oriental | 780 | 33.8 | 0.26 | 0.534 |
|  | Mapiripán | Llanos | 12,018 | -359.4 | -0.76 | 0.011 |
|  | Mesetas | Cordillera Oriental | 1,972 | -0.6 | 0.00 | 0.992 |
|  | Puerto Concordia | Llanos | 1,295 | -73.1 | -0.46 | 0.176 |
|  | Puerto Gaitán | Llanos | 17,397 | -198.5 | -0.84 | 0.002 |
|  | Puerto Lleras | Llanos | 2,537 | -71.6 | -0.22 | 0.550 |
|  | Puerto López | Llanos | 6,985 | -57.9 | -0.26 | 0.476 |
|  | Puerto Rico | Apure-Villavicencio | 3,353 | 277.1 | 0.52 | 0.127 |
|  | Restrepo | Cordillera Oriental | 331 | 19.0 | 0.75 | 0.054 |
|  | San Carlos de Guaroa | Llanos | 806 | 108.2 | 0.78 | 0.008 |
|  | San Juan de Arama | Apure-Villavicencio | 1,188 | 16.5 | 0.43 | 0.214 |
|  | San Juanito | Cordillera Oriental | 234 | 12.5 | 0.20 | 0.585 |
|  | San Martín | Llanos | 6,023 | 10.1 | 0.04 | 0.918 |
|  | Uribe | Cordillera Oriental | 6,206 | -143.8 | -0.25 | 0.511 |
|  | Villavicencio | Apure-Villavicencio | 1,328 | 24.8 | 0.40 | 0.333 |
|  | Vista Hermosa | Cordillera Oriental | 4,911 | 140.3 | 0.60 | 0.066 |
| **Nariño** | Albán | Northwestern Andean | 46 | 9.9 | 0.66 | 0.037 |
|  | Ancuya | Northwestern Andean | 68 | 0.1 | 0.45 | 0.193 |
|  | Arboleda | Northwestern Andean | 62 | 2.2 | 0.53 | 0.113 |
|  | Barbacoas | Northwestern Andean | 2,531 | 408.3 | 0.48 | 0.159 |
|  | Belén | Northwestern Andean | 38 | 1.1 | 0.55 | 0.102 |
|  | Buesaco | Northwestern Andean | 651 | 12.6 | 0.30 | 0.401 |
|  | Chachaguí | Northwestern Andean | 145 | 14.5 | 0.77 | 0.010 |
|  | Colón | Northwestern Andean | 64 | 8.7 | 0.59 | 0.074 |
|  | Consaca | Northwestern Andean | 127 | 5.9 | 0.40 | 0.247 |
|  | Contadero | Northwestern Andean | 42 | 1.4 | 0.49 | 0.148 |
|  | Córdoba | Northern Andean | 276 | 21.2 | 0.38 | 0.357 |
|  | Cumbal | Northwestern Andean | 1,171 | 50.5 | 0.26 | 0.461 |
|  | Cumbitara | Northwestern Andean | 390 | 29.8 | 0.63 | 0.092 |
|  | El Charco | Chocó-Darién Moist | 2,308 | 348.2 | 0.60 | 0.069 |
|  | El Peñol | Magdalena Valley Dry | 127 | 0.8 | 0.24 | 0.503 |
|  | El Rosario | Northwestern Andean | 616 | 113.9 | 0.56 | 0.094 |
|  | El Tablón de Gómez | Northwestern Andean | 270 | 36.3 | 0.75 | 0.013 |
|  | El Tambo | Northwestern Andean | 251 | 6.4 | 0.52 | 0.123 |
|  | Francisco Pizarro | Chocó-Darién Moist | 595 | -14.2 | -0.11 | 0.817 |
|  | Funes | Northwestern Andean | 386 | 65.9 | 0.98 | 0.118 |
|  | Guachucal | Northwestern Andean | 150 | 0.2 | 0.09 | 0.805 |
|  | Gualmatán | Northwestern Andean | 33 | 2.2 | 0.54 | 0.106 |
|  | Iles | Northwestern Andean | 77 | 1.8 | 0.47 | 0.171 |
|  | Ipiales | Northern Andean | 1,580 | 171.1 | 0.88 | 0.116 |
|  | La Cruz | Northwestern Andean | 223 | 2.6 | 0.32 | 0.371 |
|  | La Florida | Northwestern Andean | 132 | 11.1 | 0.77 | 0.010 |
|  | La Llanada | Northwestern Andean | 306 | 48.5 | 0.50 | 0.138 |
|  | La Tola | Chocó-Darién Moist | 380 | -41.8 | -0.40 | 0.254 |
|  | La Unión | Northwestern Andean | 148 | 7.9 | 0.54 | 0.105 |
|  | Leiva | Northwestern Andean | 302 | 20.3 | 0.43 | 0.210 |
|  | Linares | Northwestern Andean | 139 | 9.1 | 0.70 | 0.024 |
|  | Los Andes | Northwestern Andean | 853 | 106.9 | 0.43 | 0.338 |
|  | Maguí | Chocó-Darién Moist | 1,634 | 393.6 | 0.66 | 0.038 |
|  | Mallama | Northwestern Andean | 498 | 31.6 | 0.51 | 0.128 |
|  | Mosquera | Chocó-Darién Moist | 614 | -25.1 | -0.20 | 0.744 |
|  | Nariño | Northwestern Andean | 53 | 0.6 | 0.24 | 0.511 |
|  | Olaya Herrera | Chocó-Darién Moist | 870 | -40.5 | -0.17 | 0.646 |
|  | Ospina | Northwestern Andean | 63 | 0.1 | 0.75 | 0.013 |
|  | Pasto | Northwestern Andean | 1,010 | 36.3 | 0.26 | 0.501 |
|  | Policarpa | Northwestern Andean | 427 | 70.0 | 0.62 | 0.056 |
|  | Potosi | Northwestern Andean | 295 | 30.7 | 0.74 | 0.153 |
|  | Providencia | Northwestern Andean | 38 | 2.6 | 0.46 | 0.186 |
|  | Puerres | Northern Andean | 320 | 56.7 | 0.99 | 0.010 |
|  | Pupiales | Northwestern Andean | 127 | 1.3 | 0.37 | 0.289 |
|  | Ricaurte | Northwestern Andean | 1,483 | 159.9 | 0.37 | 0.418 |
|  | Roberto Payán | Chocó-Darién Moist | 1,260 | 209.9 | 0.53 | 0.112 |
|  | Samaniego | Northwestern Andean | 578 | 121.3 | 0.59 | 0.095 |
|  | San Bernardo | Northwestern Andean | 66 | 11.2 | 0.70 | 0.023 |
|  | San Lorenzo | Northwestern Andean | 251 | 10.8 | 0.45 | 0.187 |
|  | San Pablo | Northwestern Andean | 117 | 2.8 | 0.65 | 0.041 |
|  | San Pedro de Cartago | Northwestern Andean | 57 | 2.3 | 0.49 | 0.146 |
|  | Sandona | Northwestern Andean | 94 | 4.5 | 0.34 | 0.331 |
|  | Santa Bárbara | Chocó-Darién Moist | 1,140 | 81.7 | 0.26 | 0.492 |
|  | Santacruz | Northwestern Andean | 512 | 87.7 | 0.69 | 0.028 |
|  | Sapuyes | Northwestern Andean | 127 | 4.0 | 0.64 | 0.046 |
|  | Taminango | Magdalena Valley Dry | 233 | 0.2 | 0.14 | 0.690 |
|  | Tangua | Northwestern Andean | 222 | -0.9 | -0.07 | 0.848 |
|  | Tumaco | Chocó-Darién Moist | 3,831 | 21.2 | 0.04 | 0.927 |
|  | Túquerres | Northwestern Andean | 212 | 3.3 | 0.71 | 0.022 |
|  | Yacuanquer | Northwestern Andean | 109 | 0.9 | 0.19 | 0.600 |
| **Norte Santander** | Abrego | Cordillera Oriental | 1,398 | -10.9 | -0.13 | 0.723 |
|  | Arboledas | Cordillera Oriental | 468 | 7.4 | 0.08 | 0.824 |
|  | Bochalema | Cordillera Oriental | 191 | 8.8 | 0.17 | 0.638 |
|  | Bucarasica | Cordillera Oriental | 271 | 40.0 | 0.51 | 0.132 |
|  | Cáchira | Northern Andean | 627 | 32.0 | 0.38 | 0.277 |
|  | Cácota | Cordillera Oriental | 130 | -0.5 | -0.17 | 0.640 |
|  | Chinacota | Cordillera Oriental | 163 | 17.8 | 0.40 | 0.254 |
|  | Chitagá | Cordillera Oriental | 1,185 | -72.4 | -0.55 | 0.205 |
|  | Convención | Cordillera Oriental | 964 | -21.7 | -0.28 | 0.439 |
|  | Cúcuta | Mag-Urabá Moist | 1,147 | 50.9 | 0.46 | 0.180 |
|  | Cucutilla | Cordillera Oriental | 366 | 23.4 | 0.40 | 0.248 |
|  | Durania | Cordillera Oriental | 173 | 24.4 | 0.41 | 0.242 |
|  | El Carmen | Cordillera Oriental | 1,692 | 83.4 | 0.47 | 0.165 |
|  | El Tarra | Mag-Urabá Moist | 726 | 85.9 | 0.64 | 0.047 |
|  | El Zulia | Mag-Urabá Moist | 517 | 42.6 | 0.73 | 0.017 |
|  | Gramalote | Cordillera Oriental | 146 | 11.9 | 0.41 | 0.243 |
|  | Hacarí | Cordillera Oriental | 420 | 4.0 | 0.05 | 0.889 |
|  | Herrán | Cordillera Oriental | 117 | -0.2 | -0.02 | 0.958 |
|  | La Esperanza | Mag-Urabá Moist | 663 | 1.7 | 0.03 | 0.939 |
|  | La Playa | Cordillera Oriental | 254 | 17.4 | 0.42 | 0.227 |
|  | Labateca | Cordillera Oriental | 275 | -18.5 | -0.37 | 0.291 |
|  | Los Patios | Cordillera Oriental | 130 | 13.9 | 0.53 | 0.117 |
|  | Lourdes | Cordillera Oriental | 97 | 10.9 | 0.34 | 0.330 |
|  | Mutiscua | Cordillera Oriental | 157 | 1.9 | 0.32 | 0.364 |
|  | Ocaña | Northern Andean | 657 | 7.5 | 0.19 | 0.602 |
|  | Pamplona | Cordillera Oriental | 307 | 9.4 | 0.34 | 0.331 |
|  | Pamplonita | Cordillera Oriental | 164 | 19.0 | 0.45 | 0.187 |
|  | Puerto Santander | Mag-Urabá Moist | 46 | 1.4 | 0.63 | 0.052 |
|  | Ragonvalía | Cordillera Oriental | 104 | 2.4 | 0.16 | 0.664 |
|  | Salázar | Cordillera Oriental | 498 | -28.6 | -0.24 | 0.504 |
|  | San Calixto | Cordillera Oriental | 397 | 15.7 | 0.19 | 0.592 |
|  | San Cayetano | Mag-Urabá Moist | 145 | 23.3 | 0.59 | 0.072 |
|  | Santiago | Cordillera Oriental | 183 | 19.7 | 0.63 | 0.050 |
|  | Sardinata | Mag-Urabá Moist | 1,454 | 211.3 | 0.78 | 0.008 |
|  | Silos | Northern Páramo | 386 | -0.7 | -0.29 | 0.408 |
|  | Teorama | Cordillera Oriental | 919 | 7.6 | 0.07 | 0.856 |
|  | Tibú | Mag-Urabá Moist | 2,680 | 638.2 | 0.88 | 0.001 |
|  | Toledo | Cordillera Oriental | 1,517 | -69.4 | -0.45 | 0.370 |
|  | Villa Caro | Cordillera Oriental | 402 | 14.3 | 0.24 | 0.509 |
|  | Villa del Rosario | Cordillera Oriental | 102 | 3.3 | 0.52 | 0.119 |
| **Putumayo** | Colón | Northern Andean | 81 | 10.3 | 0.56 | 0.091 |
|  | Mocoa | Northern Andean | 1,386 | 137.6 | 0.52 | 0.285 |
|  | Orito | Northern Andean | 2,108 | 194.7 | 0.49 | 0.261 |
|  | Puerto Asís | Caquetá Moist | 2,799 | -20.1 | -0.06 | 0.876 |
|  | Puerto Caicedo | Caquetá Moist | 843 | 22.5 | 0.09 | 0.808 |
|  | Puerto Guzmán | Caquetá Moist | 4,518 | -430.1 | -0.47 | 0.174 |
|  | Puerto Leguízamo | Caquetá Moist | 10,781 | -525.5 | -0.38 | 0.275 |
|  | San Francisco | Northern Andean | 518 | -3.3 | -0.05 | 0.931 |
|  | San Miguel | Caquetá Moist | 341 | 6.8 | 0.24 | 0.512 |
|  | Santiago | Northern Andean | 487 | 56.0 | 0.92 | 0.009 |
|  | Sibundoy | Northern Andean | 65 | 3.0 | 0.51 | 0.135 |
|  | Valle del Gamuez | Caquetá Moist | 813 | 59.7 | 0.42 | 0.231 |
|  | Villagarzón | Caquetá Moist | 1,273 | -78.1 | -0.34 | 0.507 |
| **Quindio** | Armenia | Cauca-Valley Montane | 120 | 2.9 | 0.46 | 0.186 |
|  | Buenavista | Cauca-Valley Montane | 38 | 0.7 | 0.04 | 0.910 |
|  | Calarcá | Cauca-Valley Montane | 230 | -1.4 | -0.04 | 0.938 |
|  | Circasia | Cauca-Valley Montane | 92 | 6.8 | 0.41 | 0.243 |
|  | Córdoba | Cauca-Valley Montane | 96 | -13.8 | -0.93 | 0.007 |
|  | Filandia | Cauca-Valley Montane | 106 | 0.6 | 0.04 | 0.912 |
|  | Génova | Cauca-Valley Montane | 296 | -8.7 | -0.10 | 0.774 |
|  | La Tebaida | Cauca-Valley Montane | 92 | 4.7 | 0.92 | 0.000 |
|  | Montenegro | Cauca-Valley Montane | 150 | 9.6 | 0.56 | 0.095 |
|  | Pijao | Cauca-Valley Montane | 258 | -5.8 | -0.13 | 0.740 |
|  | Quimbaya | Cauca-Valley Montane | 136 | 10.0 | 0.60 | 0.066 |
|  | Salento | Cauca-Valley Montane | 347 | 14.6 | 0.16 | 0.650 |
| **Risaralda** | Apía | Cauca-Valley Montane | 151 | 14.4 | 0.56 | 0.094 |
|  | Balboa | Cauca-Valley Montane | 121 | 16.6 | 0.72 | 0.020 |
|  | Belén de Umbría | Cauca-Valley Montane | 173 | 8.8 | 0.41 | 0.237 |
|  | Dosquebradas | Cauca-Valley Montane | 70 | -3.0 | -0.21 | 0.568 |
|  | Guática | Cauca-Valley Montane | 100 | 7.6 | 0.45 | 0.192 |
|  | La Celia | Cauca-Valley Montane | 91 | 13.1 | 0.44 | 0.201 |
|  | La Virginia | Magdalena Valley Dry | 32 | 7.9 | 0.73 | 0.016 |
|  | Marsella | Cauca-Valley Montane | 150 | 25.2 | 0.51 | 0.128 |
|  | Mistrató | Cauca-Valley Montane | 596 | 44.2 | 0.31 | 0.423 |
|  | Pereira | Cauca-Valley Montane | 600 | 15.6 | 0.38 | 0.279 |
|  | Pueblo Rico | Northwestern Andean | 633 | 90.0 | 0.44 | 0.232 |
|  | Quinchía | Cauca-Valley Montane | 142 | 1.9 | 0.15 | 0.676 |
|  | Santa Rosa de Cabal | Cauca-Valley Montane | 568 | -12.5 | -0.25 | 0.690 |
|  | Santuarío | Cauca-Valley Montane | 208 | 11.7 | 0.29 | 0.415 |
| **Santander** | Aguada | Northern Andean | 69 | 9.1 | 0.55 | 0.099 |
|  | Albania | Northern Andean | 187 | 32.3 | 0.69 | 0.027 |
|  | Aratoca | Northern Andean | 161 | 9.4 | 0.73 | 0.016 |
|  | Barbosa | Northern Andean | 50 | 0.8 | 0.31 | 0.376 |
|  | Barichara | Northern Andean | 127 | 0.5 | 0.55 | 0.098 |
|  | Barrancabermeja | Mag-Urabá Moist | 1,289 | -18.7 | -0.13 | 0.723 |
|  | Betulia | Northern Andean | 423 | 14.6 | 0.22 | 0.548 |
|  | Bolívar | Northern Andean | 1,001 | -12.1 | -0.19 | 0.602 |
|  | Bucaramanga | Northern Andean | 157 | 16.4 | 0.65 | 0.044 |
|  | Cabrera | Northern Andean | 70 | 0.2 | 0.52 | 0.122 |
|  | California | Northern Páramo | 45 | 7.8 | 0.66 | 0.037 |
|  | Capitanejo | Northern Andean | 79 | 0.0 | 0.52 | 0.122 |
|  | Carcasí | Northern Andean | 265 | 4.3 | 0.24 | 0.509 |
|  | Cepita | Northern Andean | 108 | 4.5 | 0.52 | 0.126 |
|  | Cerrito | Northern Páramo | 431 | -2.7 | -0.28 | 0.437 |
|  | Charalá | Northern Andean | 358 | 19.2 | 0.63 | 0.049 |
|  | Charta | Northern Andean | 129 | 17.6 | 0.71 | 0.021 |
|  | Chima | Northern Andean | 184 | 1.2 | 0.03 | 0.930 |
|  | Chipatá | Northern Andean | 77 | 1.5 | 0.41 | 0.239 |
|  | Cimitarra | Mag-Urabá Moist | 3,203 | -146.0 | -0.61 | 0.061 |
|  | Concepción | Northern Páramo | 347 | 5.4 | 0.50 | 0.139 |
|  | Confines | Northern Andean | 59 | 4.0 | 0.71 | 0.021 |
|  | Contratación | Northern Andean | 95 | 13.8 | 0.63 | 0.050 |
|  | Coromoro | Northern Andean | 574 | 20.8 | 0.31 | 0.385 |
|  | Curití | Northern Andean | 243 | 11.6 | 0.81 | 0.005 |
|  | El Carmen de Chucurí | Northern Andean | 906 | -22.9 | -0.18 | 0.619 |
|  | El Guacamayo | Northern Andean | 116 | 12.1 | 0.46 | 0.177 |
|  | El Peñón | Northern Andean | 398 | -11.4 | -0.21 | 0.562 |
|  | El Playón | Northern Andean | 461 | 5.9 | 0.08 | 0.828 |
|  | Encino | Northern Andean | 436 | 0.1 | 0.01 | 0.989 |
|  | Enciso | Northern Andean | 77 | 3.5 | 0.45 | 0.188 |
|  | Florián | Northern Andean | 201 | 31.8 | 0.69 | 0.028 |
|  | Floridablanca | Northern Andean | 97 | 2.9 | 0.24 | 0.502 |
|  | Galán | Northern Andean | 211 | -7.6 | -0.27 | 0.456 |
|  | Gambita | Northern Andean | 524 | 18.8 | 0.49 | 0.153 |
|  | Girón | Northern Andean | 496 | 11.6 | 0.27 | 0.458 |
|  | Guaca | Northern Andean | 272 | 14.4 | 0.42 | 0.226 |
|  | Guadalupe | Northern Andean | 196 | 10.8 | 0.62 | 0.054 |
|  | Guapota | Northern Andean | 60 | -0.5 | -0.32 | 0.368 |
|  | Guavata | Northern Andean | 76 | 4.6 | 0.29 | 0.413 |
|  | Guepsa | Northern Andean | 45 | 0.4 | 0.44 | 0.203 |
|  | Hato | Northern Andean | 190 | 0.2 | 0.00 | 0.993 |
|  | Jesus María | Northern Andean | 101 | 2.8 | 0.14 | 0.693 |
|  | Jordan | Northern Andean | 36 | 1.3 | 0.63 | 0.051 |
|  | La Belleza | Northern Andean | 286 | 28.7 | 0.72 | 0.019 |
|  | La Paz | Northern Andean | 272 | 54.0 | 0.60 | 0.066 |
|  | Landázuri | Northern Andean | 616 | -5.1 | -0.06 | 0.870 |
|  | Lebrija | Northern Andean | 540 | 66.2 | 0.68 | 0.029 |
|  | Los Santos | Northern Andean | 292 | 1.1 | 0.51 | 0.133 |
|  | Macaravita | Northern Andean | 108 | 3.0 | 0.38 | 0.274 |
|  | Málaga | Northern Andean | 57 | 1.5 | 0.30 | 0.395 |
|  | Matanza | Northern Andean | 111 | -6.4 | -0.27 | 0.445 |
|  | Mogotes | Northern Andean | 510 | 61.8 | 0.88 | 0.001 |
|  | Molagavita | Northern Andean | 176 | 8.2 | 0.62 | 0.058 |
|  | Ocamonte | Northern Andean | 54 | 3.1 | 0.87 | 0.001 |
|  | Oiba | Northern Andean | 312 | 38.3 | 0.86 | 0.002 |
|  | Onzaga | Northern Andean | 499 | 33.8 | 0.67 | 0.035 |
|  | Palmas del Socorro | Northern Andean | 63 | 0.5 | 0.19 | 0.598 |
|  | Páramo | Northern Andean | 81 | 7.1 | 0.78 | 0.008 |
|  | Piedecuesta | Northern Andean | 475 | 31.6 | 0.76 | 0.010 |
|  | Pinchote | Northern Andean | 57 | 9.8 | 0.82 | 0.004 |
|  | Puente Nacional | Northern Andean | 274 | 32.9 | 0.64 | 0.047 |
|  | Puerto Parra | Mag-Urabá Moist | 766 | -52.5 | -0.67 | 0.033 |
|  | Puerto Wilches | Mag-Urabá Moist | 1,535 | 121.4 | 0.45 | 0.189 |
|  | Rionegro | Northern Andean | 1,282 | 29.9 | 0.41 | 0.240 |
|  | Sabana de Torres | Mag-Urabá Moist | 1,456 | 47.9 | 0.26 | 0.468 |
|  | San Andrés | Northern Andean | 289 | 25.6 | 0.68 | 0.031 |
|  | San Benito | Northern Andean | 60 | 0.8 | 0.61 | 0.061 |
|  | San Gil | Northern Andean | 157 | 6.5 | 0.62 | 0.055 |
|  | San Joaquín | Northern Andean | 146 | 27.2 | 0.84 | 0.002 |
|  | San José de Miranda | Northern Andean | 74 | 1.1 | 0.55 | 0.101 |
|  | San Miguel | Northern Andean | 69 | 2.7 | 0.41 | 0.240 |
|  | San Vicente de Chucuri | Northern Andean | 1,101 | -43.4 | -0.22 | 0.551 |
|  | Santa Bárbara | Northern Andean | 188 | 19.0 | 0.62 | 0.055 |
|  | Santa Elena del Opón | Northern Andean | 356 | 27.4 | 0.36 | 0.311 |
|  | Simacota | Northern Andean | 960 | -28.8 | -0.28 | 0.431 |
|  | Socorro | Northern Andean | 125 | 15.2 | 0.85 | 0.002 |
|  | Suaita | Northern Andean | 261 | 14.8 | 0.59 | 0.074 |
|  | Sucre | Northern Andean | 548 | 16.0 | 0.28 | 0.438 |
|  | Surata | Northern Andean | 376 | 44.2 | 0.81 | 0.005 |
|  | Tona | Northern Páramo | 338 | 32.7 | 0.72 | 0.019 |
|  | Valle de San Jose | Northern Andean | 91 | 4.0 | 0.43 | 0.210 |
|  | Vélez | Northern Andean | 473 | 23.6 | 0.35 | 0.317 |
|  | Vetas | Northern Páramo | 91 | 6.5 | 0.69 | 0.026 |
|  | Zapatoca | Northern Andean | 370 | 5.5 | 0.29 | 0.419 |
| **Sucre** | Caimito | Mag-Urabá Moist | 425 | 14.6 | 0.74 | 0.015 |
|  | Chalan | Guajira Xeric | 85 | 3.8 | 0.15 | 0.685 |
|  | Coloso | Guajira Xeric | 126 | 1.6 | 0.05 | 0.883 |
|  | Coveñas | Guajira Xeric | 55 | 0.4 | 0.24 | 0.601 |
|  | El Roble | Mag-Urabá Moist | 213 | -0.1 | -0.17 | 0.631 |
|  | Galeras | Mag-Urabá Moist | 296 | 0.0 | -0.17 | 0.631 |
|  | Guaranda | Mag-Urabá Moist | 373 | 4.9 | 0.21 | 0.560 |
|  | La Unión | Mag-Urabá Moist | 223 | 0.0 | 0.04 | 0.917 |
|  | Los Palmitos | Mag-Urabá Moist | 212 | 5.3 | 0.44 | 0.204 |
|  | Majagual | Mag-Urabá Moist | 950 | 55.1 | 0.53 | 0.119 |
|  | Morroa | Guajira Xeric | 175 | 5.0 | 0.63 | 0.050 |
|  | Ovejas | Mag-Urabá Moist | 472 | 29.7 | 0.69 | 0.029 |
|  | Palmito | Guajira Xeric | 173 | 0.1 | 0.16 | 0.668 |
|  | Sampués | Mag-Urabá Moist | 197 | 0.0 | -0.26 | 0.466 |
|  | San Benito Abad | Mag-Urabá Moist | 1,521 | 164.3 | 0.83 | 0.003 |
|  | San Luis de Since | Mag-Urabá Moist | 462 | 0.0 | 0.17 | 0.631 |
|  | San Marcos | Mag-Urabá Moist | 967 | 39.0 | 0.90 | 0.000 |
|  | San Onofre | Guajira Xeric | 1,052 | 45.9 | 0.75 | 0.012 |
|  | Santiago de Tolú | Guajira Xeric | 318 | 4.9 | 0.63 | 0.066 |
|  | Sincelejo | Mag-Urabá Moist | 291 | 0.0 | -0.17 | 0.631 |
|  | Sucre | Mag-Urabá Moist | 1,110 | 112.3 | 0.64 | 0.065 |
|  | Tolu viejo | Guajira Xeric | 283 | 2.7 | 0.31 | 0.390 |
| **Tolima** | Alpujarra | Northern Andean | 538 | 6.6 | 0.48 | 0.158 |
|  | Alvarado | Magdalena Valley Dry | 345 | 0.8 | 0.02 | 0.947 |
|  | Ambalema | Magdalena Valley Dry | 229 | 5.5 | 0.15 | 0.687 |
|  | Anzoátegui | Northern Andean | 480 | 6.6 | 0.19 | 0.608 |
|  | Armero | Magdalena Valley Dry | 444 | 2.3 | 0.03 | 0.933 |
|  | Ataco | Northern Andean | 1,040 | 78.5 | 0.48 | 0.156 |
|  | Cajamarca | Northern Andean | 514 | 22.1 | 0.20 | 0.602 |
|  | Carmen de Apicalá | Magdalena Valley Dry | 192 | 2.8 | 0.07 | 0.855 |
|  | Casabianca | Northern Andean | 185 | -5.0 | -0.15 | 0.684 |
|  | Chaparral | Northern Andean | 2,104 | 140.4 | 0.52 | 0.231 |
|  | Coello | Magdalena Valley Dry | 336 | -18.4 | -0.21 | 0.559 |
|  | Coyaima | Magdalena Valley Dry | 675 | 15.2 | 0.26 | 0.473 |
|  | Cunday | Magdalena Valley Dry | 486 | -17.6 | -0.17 | 0.631 |
|  | Dolores | Northern Andean | 593 | 18.8 | 0.65 | 0.042 |
|  | Espinal | Magdalena Valley Dry | 215 | -10.4 | -0.27 | 0.452 |
|  | Falán | Magdalena Valley Dry | 174 | -19.6 | -0.32 | 0.368 |
|  | Flandes | Magdalena Valley Dry | 102 | -3.9 | -0.62 | 0.056 |
|  | Fresno | Northern Andean | 229 | -30.4 | -0.71 | 0.021 |
|  | Guamo | Magdalena Valley Dry | 512 | -13.1 | -0.28 | 0.425 |
|  | Herveo | Northern Andean | 348 | -10.8 | -0.28 | 0.462 |
|  | Honda | Magdalena Valley Dry | 314 | -6.3 | -0.11 | 0.766 |
|  | Ibagué | Northern Andean | 1,405 | 9.8 | 0.06 | 0.881 |
|  | Icononzo | Northern Andean | 216 | 3.9 | 0.22 | 0.672 |
|  | Lérida | Magdalena Valley Dry | 273 | -13.9 | -0.43 | 0.216 |
|  | Líbano | Northern Andean | 287 | -15.1 | -0.37 | 0.295 |
|  | Mariquita | Magdalena Valley Dry | 278 | -42.5 | -0.44 | 0.200 |
|  | Melgar | Magdalena Valley Dry | 192 | 4.0 | 0.07 | 0.846 |
|  | Murillo | Northern Andean | 428 | 2.1 | 0.17 | 0.631 |
|  | Natagaima | Magdalena Valley Dry | 872 | 17.7 | 0.22 | 0.545 |
|  | Ortega | Magdalena Valley Dry | 951 | 13.9 | 0.10 | 0.792 |
|  | Palocabildo | Northern Andean | 65 | -4.5 | -0.48 | 0.161 |
|  | Piedras | Magdalena Valley Dry | 346 | -4.6 | -0.14 | 0.709 |
|  | Planadas | Northern Andean | 1,677 | 32.6 | 0.20 | 0.674 |
|  | Prado | Magdalena Valley Dry | 420 | -33.1 | -0.51 | 0.136 |
|  | Purificación | Magdalena Valley Dry | 430 | -26.4 | -0.55 | 0.097 |
|  | Ríoblanco | Northern Andean | 2,012 | -38.8 | -0.17 | 0.785 |
|  | Roncesvalles | Northern Andean | 793 | 24.0 | 0.23 | 0.581 |
|  | Rovira | Northern Andean | 708 | 40.2 | 0.42 | 0.353 |
|  | Saldaña | Magdalena Valley Dry | 207 | -3.9 | -0.65 | 0.043 |
|  | San Antonio | Northern Andean | 389 | 74.2 | 0.75 | 0.019 |
|  | San Luis | Magdalena Valley Dry | 427 | -14.2 | -0.21 | 0.565 |
|  | Santa Isabel | Northern Andean | 277 | -0.1 | -0.03 | 0.929 |
|  | Suárez | Magdalena Valley Dry | 192 | -4.4 | -0.19 | 0.597 |
|  | Valle de San Juan | Magdalena Valley Dry | 193 | -22.9 | -0.50 | 0.145 |
|  | Venadillo | Magdalena Valley Dry | 330 | -26.5 | -0.53 | 0.116 |
|  | Villahermosa | Northern Andean | 283 | 2.0 | 0.06 | 0.872 |
|  | Villarrica | Northern Andean | 456 | 60.9 | 0.95 | 0.194 |
| **Valle del Cauca** | Alcalá | Cauca-Valley Montane | 61 | 1.7 | 0.39 | 0.267 |
|  | Andalucía | Cauca-Valley Montane | 158 | 5.3 | 0.51 | 0.305 |
|  | Ansermanuevo | Cauca-Valley Montane | 351 | 20.8 | 0.41 | 0.237 |
|  | Argelia | Cauca-Valley Montane | 85 | 26.8 | 0.65 | 0.043 |
|  | Bolívar | Northwestern Andean | 796 | 196.7 | 0.74 | 0.093 |
|  | Buenaventura | Chocó-Darién Moist | 6,573 | 203.5 | 0.26 | 0.527 |
|  | Buga | Cauca-Valley Montane | 850 | -13.3 | -0.34 | 0.507 |
|  | Bugalagrande | Cauca-Valley Montane | 453 | 22.0 | 0.71 | 0.021 |
|  | Caicedonia | Cauca-Valley Montane | 151 | 0.2 | 0.03 | 0.952 |
|  | Cali | Cauca-Valley Montane | 536 | -23.0 | -0.38 | 0.530 |
|  | Candelaria | Magdalena Valley Dry | 298 | 5.2 | 0.24 | 0.503 |
|  | Cartago | Magdalena Valley Dry | 243 | 12.5 | 0.89 | 0.000 |
|  | Dagua | Northwestern Andean | 1,004 | 72.9 | 0.34 | 0.512 |
|  | El Águila | Cauca-Valley Montane | 245 | -5.7 | -0.09 | 0.798 |
|  | El Cairo | Northwestern Andean | 295 | 9.3 | 0.23 | 0.666 |
|  | El Cerrito | Cauca-Valley Montane | 444 | 31.5 | 0.68 | 0.065 |
|  | El Dovio | Northwestern Andean | 314 | 66.3 | 0.56 | 0.323 |
|  | Florida | Cauca-Valley Montane | 414 | -4.3 | -0.09 | 0.887 |
|  | Ginebra | Cauca-Valley Montane | 269 | -12.7 | -0.37 | 0.759 |
|  | Guacarí | Magdalena Valley Dry | 163 | 19.8 | 0.66 | 0.055 |
|  | Jamundí | Cauca-Valley Montane | 589 | -21.2 | -0.34 | 0.372 |
|  | La Cumbre | Northwestern Andean | 207 | 4.4 | 0.38 | 0.275 |
|  | La Unión | Magdalena Valley Dry | 117 | 0.5 | 0.76 | 0.011 |
|  | La Victoria | Cauca-Valley Montane | 262 | 17.3 | 0.65 | 0.042 |
|  | Obando | Magdalena Valley Dry | 203 | 13.2 | 0.60 | 0.068 |
|  | Palmira | Cauca-Valley Montane | 1,020 | 33.1 | 0.40 | 0.258 |
|  | Pradera | Cauca-Valley Montane | 359 | 0.9 | 0.02 | 0.972 |
|  | Restrepo | Northwestern Andean | 104 | 3.2 | 0.46 | 0.179 |
|  | Riofrío | Cauca-Valley Montane | 301 | 2.6 | 0.10 | 0.828 |
|  | Roldanillo | Magdalena Valley Dry | 204 | 4.6 | 0.88 | 0.001 |
|  | San Pedro | Cauca-Valley Montane | 192 | 17.1 | 0.62 | 0.135 |
|  | Sevilla | Cauca-Valley Montane | 568 | -3.8 | -0.06 | 0.896 |
|  | Toro | Cauca-Valley Montane | 181 | 8.1 | 0.50 | 0.138 |
|  | Trujillo | Cauca-Valley Montane | 231 | -1.6 | -0.13 | 0.811 |
|  | Tuluá | Cauca-Valley Montane | 795 | -13.1 | -0.17 | 0.722 |
|  | Ulloa | Cauca-Valley Montane | 38 | 2.0 | 0.41 | 0.237 |
|  | Versalles | Northwestern Andean | 466 | -21.7 | -0.23 | 0.585 |
|  | Vijes | Northwestern Andean | 118 | 4.5 | 0.57 | 0.086 |
|  | Yotoco | Cauca-Valley Montane | 377 | 13.5 | 0.59 | 0.072 |
|  | Yumbo | Cauca-Valley Montane | 232 | 3.3 | 0.17 | 0.681 |
|  | Zarzal | Magdalena Valley Dry | 355 | 9.5 | 0.81 | 0.005 |
| **Vaupes** | Caruru | Caquetá Moist | 6,758 | 78.8 | 0.26 | 0.464 |
|  | Mitu | Caquetá Moist | 16,558 | -23.4 | -0.09 | 0.809 |
|  | Pacoa | Caquetá Moist | 13,694 | 32.1 | 0.14 | 0.707 |
|  | Papunaua | Caquetá Moist | 5,407 | 20.1 | 0.11 | 0.768 |
|  | Taraira | Caquetá Moist | 6,577 | -14.9 | -0.07 | 0.841 |
|  | Yavarate | Caquetá Moist | 4,689 | -16.0 | -0.18 | 0.623 |
| **Vichada** | Cumaribo | Caquetá Moist | 65,568 | 1064.6 | 0.80 | 0.006 |
|  | La Primavera | Llanos | 17,900 | -101.1 | -0.45 | 0.191 |
|  | Puerto Carreño | Llanos | 12,288 | -126.2 | -0.49 | 0.148 |
|  | Santa Rosalía | Llanos | 3,884 | -20.1 | -0.51 | 0.129 |

Thirty three municipalities were not included because they did not have any *woody* vegetation.
